# MUM, a maternal unknown message, inhibits early establishment of the medio-lateral axis in the embryo of the kelp *Saccharina latissima*

**DOI:** 10.1242/dev.202732

**Published:** 2024-09-13

**Authors:** Samuel Boscq, Bernard Billoud, Ioannis Theodorou, Tanweer Joemmanbaks, Tanguy Dufourt, Bénédicte Charrier

**Affiliations:** ^1^ Morphogenesis of Macro Algae, UMR8227, CNRS - Sorbonne University, Station Biologique de Roscoff, Place Georges Teissier, 29680 Roscoff, France; ^2^ Institut de Génomique Fonctionnelle de Lyon (IGFL), UMR5242, ENS-Lyon, CNRS, INRAE, UCBL, 32-34 Avenue Tony Garnier, 69007 Lyon, France

**Keywords:** Algae, Body axes, Cell division orientation, Embryogenesis, Maternal signalling, Tissue patterning

## Abstract

Brown algae are multicellular photosynthetic organisms that have evolved independently of plants and other algae. Here, we have studied the determinism of body axis formation in the kelp *Saccharina latissima*. After microdissection of the embryo, we show that the stalk, an empty cell that retains the embryo on the maternal tissue, represses longitudinal cell divisions in the early embryo, thereby reinforcing the establishment of the initial apico-basal axis. In addition, it promotes cell growth and controls cell shape and arrangement in the flat oblong embryo composed of cells aligned in rows and columns. Although the stalk persists for several weeks until the embryo reaches at least 500 cells, proper embryogenesis requires connection to maternal tissue only during the first 4 days after fertilisation, i.e. before the embryo reaches the 8-cell stage. Transplantation experiments indicate that the maternal signal is not diffused in seawater, but requires contact between the embryo and the maternal tissue. This first global quantitative study of brown algal embryogenesis highlights the role of MUM, an unknown maternal message, in the control of growth axes and tissue patterning in kelp embryos.

## Teaser

**Summary:** A global quantitative study of brown algal embryogenesis highlights the role of MUM, an unknown maternal message, in the control of growth axes and tissue patterning in kelp embryos.

## INTRODUCTION

In all organisms, the establishment of spatial axes during embryonic development serves as the fundamental basis for organising the developing body. Despite the immense morphological diversity exhibited by eukaryotes, the majority of organisms can still be characterised by three primary body axes (Anlas and Trivedi, 2021). These axes can be defined based on morphological features (Barthélémy and Caraglio, 2007; Deline et al., 2018; Martinez et al., 2016) or by the presence of molecular gradients within the embryo (Friml et al., 2003; Simsek and Özbudak, 2022). Symmetry-breaking events are crucial for establishing axes within initially homogeneous states and this process is fundamental to the facilitation of cell differentiation. However, although polarity is often associated with axis establishment, axial determination can occur independently of polarity in some symmetrical systems (Cove, 2000).

Brown algae (also named Phaeophyceae) have evolved diverse morphologies (Bogaert et al., 2013; Bringloe et al., 2020; Charrier et al., 2012), ranging from small filamentous forms (e.g. order Ectocarpales) to large multilayered parenchymatous bodies that can reach up to 40 m (e.g. order Laminariales) (Cribb, 1954). They diverged from the ancestors of other extant multicellular organisms at the root of the eukaryotic tree at least 1 billion years ago and emerged as complex multicellular organisms relatively recently, around 250 million years ago (Burki et al., 2020; Kawai et al., 2015). Being evolutionarily distinct from animals, fungi, plants and other algae, brown algae possess unique biological mechanisms (Charrier et al., 2019; Nagasato et al., 2022; Terauchi et al., 2015). They have evolved diverse complex embryonic patterns. For example, contrary to some land plants and metazoans, which develop inside the maternal tissue, brown alga embryos are usually free-growing. Furthermore, after the initial stages of development, they develop simple morphologies. These two features make them easier to access for imaging and manipulation, and, consequently, brown algae such as *Fucus*, *Ectocarpus* and *Dictyota* have become development models for studying polarised cell growth (Bogaert et al., 2017; Goodner and Quatrano, 1993; Rabillé et al., 2019). Reports on *Fucus* and *Dictyota* have shown that the elongation of oospheres occurs only after fertilisation by sperm, but environmental cues such as light can change symmetry, shifting the orientation of the cell before the selection of the axis (Bogaert et al., 2015; Jaffe, 1968).

**Fig. 1.**
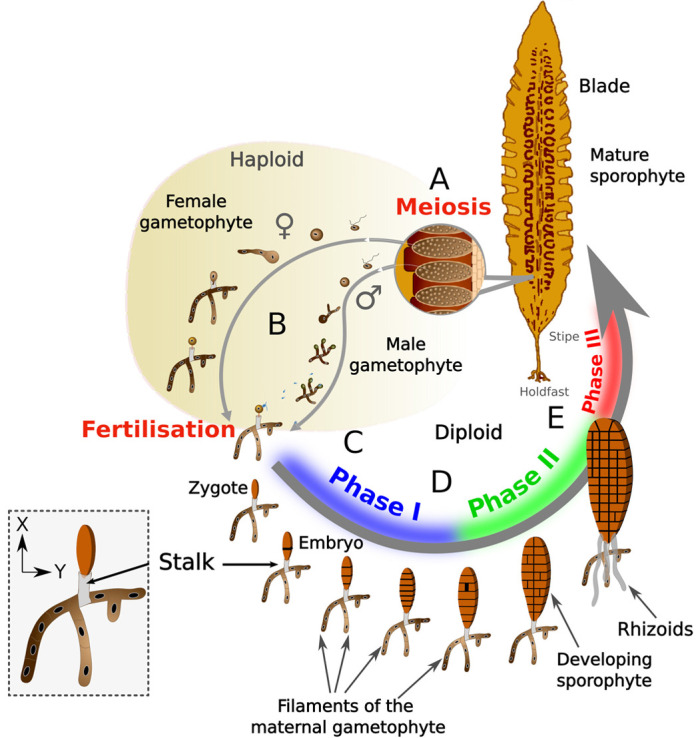
**The life cycle of *Saccharina latissima*.** (A) In mature sporophyte blades, meiosis occurs inside sporangia, and motile meiospores are released in large amounts. The spores fall on the seafloor (or plastic or glassware in the laboratory) and germinate. (B) Spores develop into filamentous female and male gametophytes. When the necessary environmental conditions are met, both types of gametophytes mature, the female gametangium (named oogonium) extrudes one egg and the male gametangium (named antheridium) releases one sperm cell. (C) After fertilisation, the embryo elongates along the *x*-axis and initially develops attached to the female gametophyte undergoing a succession of transverse, parallel divisions perpendicular to the zygote axis, up to the formation of a linear stack of eight cells (phase I in blue). (D) The embryo starts dividing longitudinally, and continues growth by alternating longitudinal and transverse cell divisions (phase II in green). (E) About 20 days after fertilisation, the embryo initiates the first divisions in the *z*-axis and undergoes cell differentiation (phase III in red). The embryo then continues to develop into a mature sporophyte. Colour codes for the three embryogenetic phases are according to Theodorou and Charrier (2023). The inset on the left better illustrates the maternal stalk.

Unlike these species, no environmental cues such as light polarisation have been reported to affect the formation of the body planes in kelps (Laminariales). However, these algae, like those of orders Desmarestiales and Sporochnales, undergo embryogenesis while being physically connected to their maternal tissue (Fig. 1) (Fritsch, 1945; Klochkova et al., 2019; Wiencke and Clayton, 1990). Therefore, they are excellent models for studying the influence of maternal factors on embryogenesis in brown algae. Although detailed studies have shed light on the impact of maternal tissues on the development of animal and land plant embryos, there is limited documentation for brown algae.

During the initial stages of development, the egg cell of *Saccharina,* a member of the Laminariales, possesses two anchored flagella that facilitate attachment to the gametangia that has differentiated from the filamentous maternal gametophyte (Fig. 1A) (Klochkova et al., 2019). After karyogamy, these flagella degrade (Fig. 1B), but the zygote firmly anchors its base to the female gametophyte, potentially through the accumulation of glycoconjugates (Klochkova et al., 2019). In 1936, Kanda was the first to report the abnormal shapes of embryos that happened to detach from the maternal gametophyte (Kanda, 1936).

Recently, the early developmental pattern of the *Saccharina latissima* sporophyte was described in three main steps, corresponding to the establishment of three distinct body planes (Theodorou and Charrier, 2023) (Fig. 1C-E). After fertilisation, the zygote elongates, followed by parallel transverse divisions that lead to the development of an 8-cell stack. Both processes – zygote elongation and transverse divisions – establish and maintain the apico-basal axis, respectively (phase I, Fig. 1C). Subsequently, the embryo undergoes two-dimensional growth, forming the medio-lateral axis, and growth in these two axes, the apico-basal axis on the one hand and the medio-lateral axis on the other hand, forms a small cellular monolayered lamina (also named the blade) (phase II, Fig. 1D). The transition to three-dimensional growth and cellular tissue differentiation (phase III, Fig. 1E) begins once the blade reaches around 800-1000 cells (Theodorou and Charrier, 2023).

Here, we present a detailed analysis of how the maternal gametophyte controls the establishment of the medio-lateral axis in the early development of the *S. latissima* sporophyte. Using microdissection to separate the maternal gametophyte from the sporophytic embryo, we monitored the development of the early embryos over time. Image segmentation followed by quantitative analyses of the morphological traits made it possible to assess the role of the maternal tissue in the control of body plane formation and cell growth in the very early stages of embryogenesis. In these experiments, we show that the physical link between the embryo and the maternal body is essential for the formation of a marked apico-basal axis, which is one of the main characteristics of adult kelps. A negative control of longitudinal divisions appears to be the mechanism through which this axis is established.

## RESULTS

To comprehensively investigate the role of maternal tissue in the growth of the embryo, we mechanically detached embryos from the stalk of the female gametophyte at different developmental stages and monitored embryogenesis for up to 14 days in standard culture conditions. Using a micro-needle, we separated the embryo from the maternal tissue in phase I at the egg (E_0_), zygote (E_1_), 2-cell (E_2_), 4-cell (E_4_) and 8-cell (E_8_) stages, and in phase II [PhII, corresponding to embryos at stages greater than the eight cells; see Theodorou and Charrier (2023) for the definition of the embryogenetic phases] by cutting the maternal stalk that physically links the embryo to the female gametophyte, leaving some pieces of cell wall still attached to the egg, zygote or embryo (hereafter named E/Z/E) (Fig. S1, see also Movie 2). The impact of the separation of the E/Z/E from the maternal tissue was then observed at a later stage, when the embryo reached embryogenic phase II, corresponding to the initiation of growth simultaneously in the longitudinal and lateral directions.

### Severing the embryo from the maternal stalk impairs both growth and the organisation of the embryo

We observed a wide range of morphological alterations, that we classified into three main groups. First, about 16% (at most) of embryos either immediately ceased developing or perished within five days post microdissection. We assumed that these embryos were severely damaged by the manipulation and we therefore excluded them from the following analyses because wounds and wound repair would likely bias the interpretation of the morphological response to stalk microdissection.

**Fig. 2.**
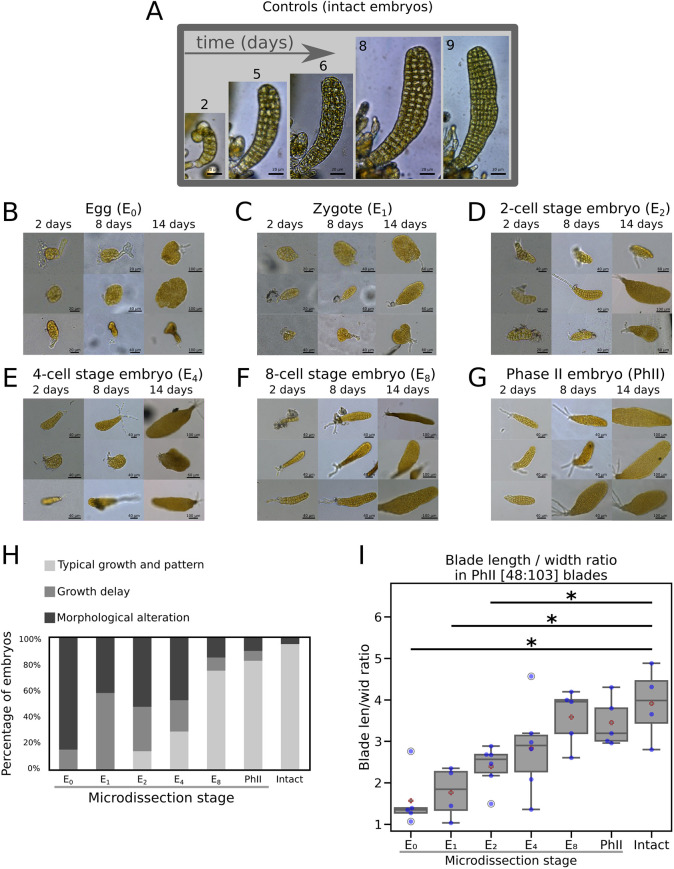
**Qualitative impact of severing the embryo from the maternal stalk.** (A-G) Comparison of morphologies in control embryos (A) with growing embryos after being severed from the stalk by microdissection at the egg (E_0_) (B), zygote (E_1_) (C), 2-cell (E_2_) (D), 4-cell (E_4_) (E), 8-cell (E_8_) (F) and early phase II (PhII) (G) stage, and observed 2, 8 and 14 days after microdissection. Three embryos illustrate the representative morphologies obtained for each microdissection time point. The number of microdissected embryos was *n*=4 (A), 100 (B), 100 (C), 80 (D), 80 (E), 40 (F) and 40 (G). (H) Stacked histogram showing the percentage of each class of developmental response: typical growth and pattern, growth delay and ‘morphological alteration’, relative to the most representative developmental pattern of intact embryos as displayed in A. In intact embryos, growth delay was set arbitrarily to 0, to be used as a relative reference. The percentage of morphological alterations observed in the population of intact embryos is indicated (see text for explanation). (I) Length/width (l/w) ratio of embryo blade (lamina) observed in phase II (48 to 103 cells) after microdissection of the maternal stalk at different developmental stages (*x*-axis). The thick red outlined plus sign shows the mean; the middle line is the median (2nd quartile); the box includes values from the 1st (25%) to the 3rd quartiles (75%); the whiskers show the extent of observations around these values, up to 1.5 times the difference between the 1st and 3rd quartiles. *P<0.05 [Wilcoxon test between ablated embryos and control (intact) embryos].

The remaining living and growing embryos displayed a range of morphogenetic responses illustrated in Fig. 2. Compared with intact embryos (Fig. 2A), those dissected from the stalk before the first cell division (egg E_0_, Fig. 2B; zygote E_1_, Fig. 2C) displayed the strongest responses. At the egg and zygote stages, all the embryos showed either delayed growth or altered morphology, which corresponds to the second and third main classes of observed developmental responses to microdissection. Delay in growth was assessed qualitatively from the observed growth rate of intact embryos (in which growth delay was arbitrarily set to 0 to serve as a reference). We noticed that the growth delays were variable among the groups of microdissected embryos (Fig. 2H and Table S1A). However, a general trend emerged of high frequencies of embryos with reduced growth for early-stage microdissection compared with later stage microdissection (58% of delayed growth for microdissected E_1_ zygotes, but only 7.5% for microdissected phase II embryos). The percentage of embryos displaying morphological alterations (third class of developmental alterations) was also higher when the stalk was removed at the egg stage (e.g. 85% of round embryos in E_0;_ Fig. 2B,H and Table S1A) than at the zygote stage (42% in E_1_; Fig. 2C,H and Table S1A). Similar proportions were observed for E_2_ and E_4_ microdissected embryos, with 52.5 and 47.5% of embryos displaying morphological alterations (Fig. 2D and E, respectively, Fig. 2H; Table S1A). In the E_8_ and PhII embryos, the effect of microdissection was weak, with at least 85% of the microdissected embryos exhibiting a typical morphological pattern (Fig. 2F and G, respectively; Fig. 2H; Table S1A). The population corresponding to intact embryos also contains embryos with altered morphologies (4.78%; Fig. 2H, Table S1A), revealing some intrinsic morphological plasticity of the strains used for the crosses (see the Materials and Methods section for details on the genetic strains). This morphological ‘noise’ has already been reported in other kelp species (Druehl et al., 2005). The proportion of atypical morphologies observed in our samples was much lower compared with that observed in the microdissected samples (4.78% versus ∼50% respectively, see above); hence, we considered that the morphological defects observed in microdissected embryos most likely resulted from severing the stalk. Hence, the connection between the embryo and the stalk before the 8-cell stage seems necessary for normal growth and morphogenesis of the embryo.

To quantitatively study the impact of dissection from the maternal stalk on the morphogenesis of embryos, we monitored the development of another series of microdissected embryos for 10 days using bright-field microscopy. First, images were captured every day for 4 days, then every 2 days, and segmented manually (Fig. S2). From the segmentation images, we measured quantitative values of several morphometric parameters from the analysis of the cell outlines using in-house software (Table S2 for the blades and Table S3 for the cells) and then compared them using statistical tests (see Materials and Methods for details; Table S4). However, before analysing the data, we calculated the probability of picking a microdissected E/Z/E displaying a morphological alteration due to the intrinsic morphological plasticity to the strain (rather than to microdissection). Based on the 4.78% of embryos with altered morphology observed in the intact population (Table S1A), we calculated that, in a sample of five embryos, the probability of having only normally developing embryos was 0.78, and the expected number of embryos with altered morphology was 0.24 (Table S1B). This value is sufficiently low for us to consider that the morphological alterations observed in the segmented embryos at each microdissection stage are due to the effect of microdissection and not to the intrinsic morphological plasticity of the genetic strain.

To observe how the shape of the embryo is affected by separation from the maternal tissue, we measured the length and the width of PhII embryos containing between 48 and 103 cells ([48:103]). This developmental interval, expressed in numbers of cells and not in numbers of days of growth, allowed us to disregard the delay in growth. Furthermore, neither egg extrusion nor fertilisation are synchronous in *Saccharina*; using embryo age as a reference was therefore impossible with the current protocol of embryo production (Theodorou et al., 2021).

We observed that, in contrast to the intact embryos that display a length/width (l/w) ratio of ∼3.5, reflecting their elongated shape, sectioning the stalk separating the egg from the maternal tissue resulted in embryos with a disc-like shape (l/w ratio ∼1) (Fig. 2I and Fig. S3B, centre). Furthermore, stalk removal at different stages between the egg and PhII revealed a gradient in the response: the earlier the stalk removal, the more disc-like the embryo. This morphological response is due to the concomitant modification of two morphological parameters: a reduction in growth along the longitudinal axis (blade length, Fig. S3C, centre) (2.0 times less in E_0_ compared with intact embryos; *P*<5.10^−2^) and an increase in growth along the medio-lateral axis (blade width, Fig. S3D, centre) (1.9 times more in E_0_ compared with intact embryos; not statistically confirmed). These changes in the direction of growth were accompanied by a significant reduction in the embryo surface area of up to 35% less than the intact embryos (E_0_ and E_1_; *P*<5.10^−2^; Tables S2 and S4).

These changes in l/w ratio (and to a lesser extent in surface area) were not only observed in embryos of 48-103 cells, but also in earlier and late embryos (Fig. S3B-D, left and right sides). This pattern suggests that, as in intact organisms, microdissected embryos maintain the ratio that they had at the time of separation from the maternal tissue. Thus, the presence of an intact maternal stalk in the early stages appears to be essential for a long period of embryogenesis and its absence cannot be compensated for up to at least the 100-cell stage, after which our samples become less reliable because there are fewer of them.

These results show that a signal related to the maternal stalk controls the development of the embryo. This maternal unknown message (hereafter named ‘MUM’) is most effective from the egg stage to the 8-cell stage, albeit decreasing in importance with time, and its impact on embryo morphology lasts up to the 100-cell stage.

### Severing the embryo from the maternal stalk results in altered cell shape and growth

From the manually segmented embryos (Fig. S2), we also quantified the impact of severing the stalk on cell morphometrics and tissue topology at different stages.

#### Cell growth

**Fig. 3.**
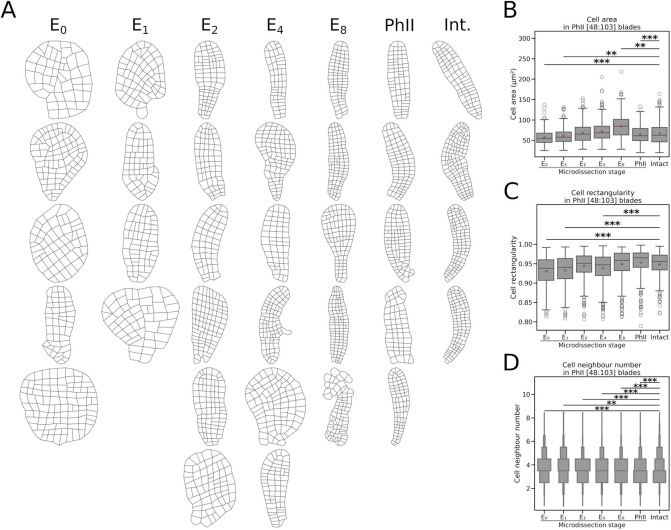
**Shape of growing embryos in response to cutting the maternal stalk*.*** The geometry of cells in phase II (PhII) embryos made up of 48 to 103 cells [48:103] was analysed using in-house blade painter software. (A) Results of manual segmentation. *n*=5 for E_0_, 4 for E_1_, 6 for E_2,_ 6 for E_4_, 5 for E_8_, 5 for PhII and 4 for control (intact). The frequency of attached embryos with altered morphology in a population of intact embryos was 0.04781 (Table S1A). From it, we estimated that, in a sample of five embryos, the probability of having only normally developing embryos was 0.78 (supplementary Materials and Methods; Table S1B), and that the expected number of embryos with altered morphology was 0.24 out of 5 (Table S1B). This figure demonstrates that the abnormal morphologies of the segmented embryos are due to the sectioning of the maternal stalk, and are not to intrinsic morphological plasticity (e.g. potentially due to parthenogenesis). (B-D) Quantitative study of cell geometry and statistical analysis. (B) Cell area (µm^2^). (C) Cell rectangularity: the shape of the cell, expressed by a rectangularity factor that assesses the level of rectangularity of a parallelepiped (cuboid cell seen in 2D). The rectangularity factor is 1 for a parallelepiped with perpendicular sides and <1 for all other cases. This factor is the lowest for parallelepipeds with very acute angles. In B and C, the thick red-outlined plus sign shows the mean; the middle line is the median (2nd quartile); the box includes values from the 1st (25%) to the 3rd quartiles (75%); the whiskers show the extent of observations around these values, up to 1.5 times the difference between the 1st and 3rd quartiles; other observations are shown as outliers (open circles). *n*=325 for E_0_, 220 for E_1_, 423 for E_2_, 415 for E_4_, 430 for E_8_, 462 for PhII and 306 for control embryos. **P<0.01; ***P<0.001 (unpaired *t*-test). (D) Number of cell neighbours. The topology of the growing embryo was studied by counting the number of cells surrounding each cell of 48- to 103-cell embryos ([48:103]). The number of neighbouring cells is plotted. Because the variable is discrete, its distribution is shown as a vertical histogram (the width of each box is proportional to the number of cells having the number of neighbours indicated in the *y*-axis), and the statistical test is a χ² test (**P<0.01; ***P<0.001).

We assessed potential alteration of cell growth by measuring cell area using in-house ‘blade_painter’ software from our sample of segmented embryos (Fig. 3A and Table S3). Cells of embryos with [48:103] cells, which were separated from the maternal tissue, displayed smaller cells, and the effect was stronger when dissection occurred early on, i.e. at the egg and zygote stages (Fig. 3B). The average cell area in intact embryos was 67.0 µm^2^, but was 57.5 µm^2^ in E_0_ embryos. Therefore, MUM controls cell size by up to 14% of the reference cell size. Plotting the value of cell area for each cell within each embryo (heatmap in Fig. S4A, middle) did not display a pattern in which cells with reduced size are localised in a specific location within the embryo. Instead, they were spread throughout the embryo, regardless of their developmental stage. Cell size reduction and their scattering throughout the embryo were two characteristics also observed in younger (developmental window: [20:47] cells) and older embryos (developmental window: [104:307] cells) (Fig. S4 left and right sides, respectively; Table S3).

**Fig. 4.**
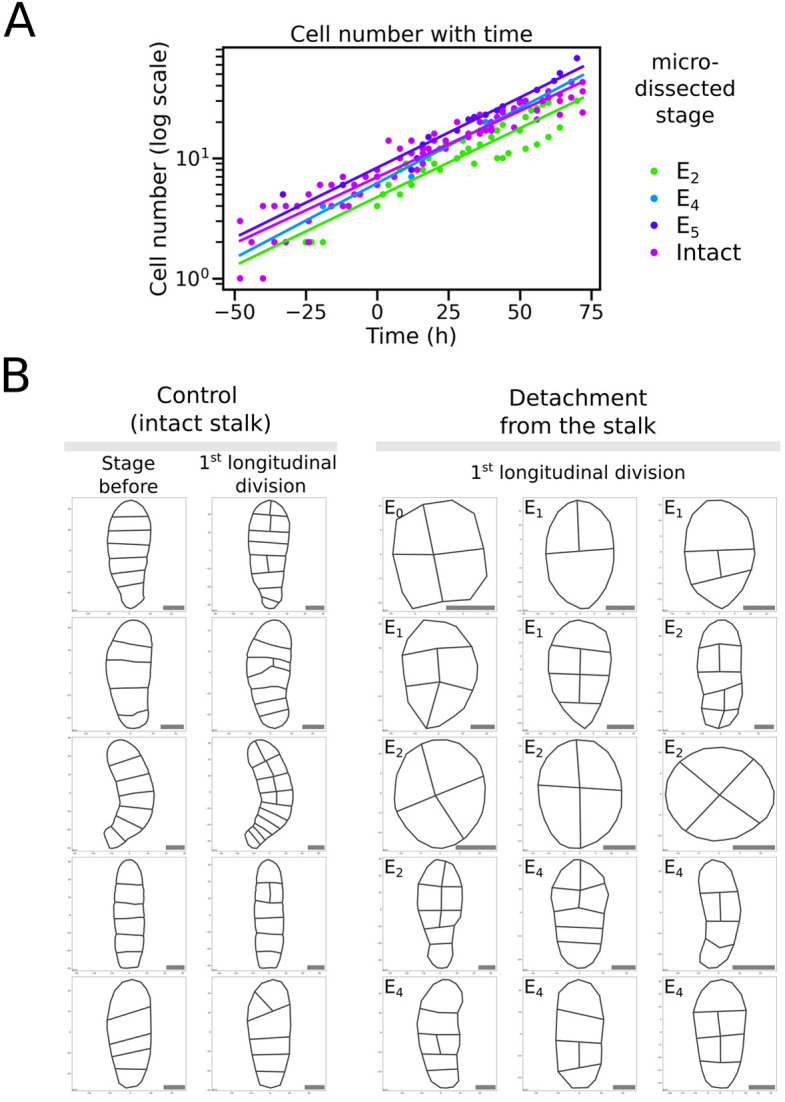
**Cell division rate and orientation of cell divisions in embryos separated from maternal tissue by microdissection*.*** (A) The number of cells of intact or microdissected embryos imaged every 2 h (as shown in Fig. S5) was plotted over time (total duration of the time-lapse: 11 days). The slope of the logarithm of this number of cells gives the growth rate. Cells divide on average every 26 h. Sample size: *n*=3 for E_2_, 1 for E_4_, 1 for E_5_ and 5 for intact. (B) The pattern of the embryo blade is displayed at the stage of the occurrence of the first longitudinal cell division(s). (Left) Patterns one time-lapse step before (left) and at the emergence of (right) the first longitudinal cell division are displayed for control organisms for which five time-lapse experiments were monitored. (Right) Pattern of segmented embryos, which were severed from maternal tissue at the E_0_, E_1_, E_2_ or E_4_ stage (stage of microdissection is indicated in the upper left-hand corner of each box). Longitudinal cell divisions were displayed using Calcofluor staining (see Materials and Methods). They took place much earlier than the 8-cell stage and often immediately after severing the stalk, except for E_0_ and E_1_, in which the first cell division was transverse or oblique (Movie 1), as in intact embryos. Scale bars: 10 μm.

We addressed whether the decrease in cell size was due to a reduction in cell growth or to a faster cell division rate. From another series of microdissected embryos (from E_2_ to E_5_) monitored with images taken every 2 h (Fig. S5), we measured the cell division rate and compared it with that of intact embryos. In these microdissected embryos, cell division took place at the same pace as in intact embryos (Fig. 4A). Cells divided every 26 h on average (from 24 h for E_4_ to 27 h for intact embryos), whether the embryo was physically attached to the maternal tissue or separated after fertilisation. As a result, we hypothesise that, in the absence of MUM, cells are smaller because they expand less while maintaining an unchanged cell division rate. Therefore, MUM appears to promote cell growth independently of cell division.

#### Cell shape and impact on neighbouring cells

We defined a rectangularity factor to quantify cell shapes in microdissected and intact embryos. The rectangularity factor was calculated based on a minimum bounding rectangle fitted around the polygonal contour of each cell. The factor equals 1 when the cell shape, taken in 2D, is a perfect rectangle (all angles joining two sides are 90°), and tends to 0 as the angles deviate from this value, corresponding to a ‘flatter’ quadrilateral or a more irregular polygon.

At the 48-103 cell stage (Table S3), we noted that cells from microdissected embryos were less rectangular than those of intact embryos (Fig. 3C). The difference was small but significant for the E_0_, E_1_ and E_4_ microdissection stages (Fig. S6B, centre). When plotted on the segmented embryos, defects in cell shape did not appear localised in specific areas of the embryos (heatmap in Fig. S6A, centre). Although cells with a shape distinct from a perfect rectangle were mainly located in the apex of the blade of intact embryos, irregular polygons were distributed randomly with no specific location within the disc-like blade of embryos microdissected at early stages (Fig. 6A). This trend was stable throughout the development of the embryos, from the 20-cell stage to the 300-cell stage (Fig. S6A and B, left and right sides, respectively). Therefore, regardless of their location, cells generally grow with altered shapes when the E/Z/E is separated from the maternal tissue before the 8-cell stage, and this impact lasts after microdissection, up to the 300-cell stage. However, this defect seems to diminish during embryo development, because microdissected embryos segmented at an earlier stage ([20:47] cells) show more irregular cell shapes, and over the entire surface of the embryo, than older embryos ([104:307] cells) (Fig. S6A,B, left-hand side compared with right-hand side).

We also studied whether this change in cell shape impacts the topology of the tissue. The number of cell neighbours was calculated for each cell in embryos microdissected at different stages, which grew to the 300-cell stage. In intact embryos, the number of cell neighbours was distributed into two main groups (Fig. 3D; heatmaps in Fig. S7A): cells at the periphery of the blade that were surrounded by three neighbours (one above, one below and one to the side) and cells located inside the blade surrounded by four neighbours (above, below and both left and right sides). In the intact embryos with 48-103 cells, 43% and 32% of cells had three and four neighbours, respectively; however, E_0_ embryos showed the reverse pattern, with, respectively 33% and 45% with three and four neighbours. A similar pattern was observed for E_1_ (31% and 37%, respectively) and E_2_ (38% and 50%, respectively) (Fig. 3D; Fig. S7B, middle; *P*<10^−2^; Table S4). The heatmap shows that these cells were localised within the lamina tissue with no specific position related to the apico-basal or medio-lateral axes (Fig. S7A, middle). Similar results were observed a few days after microdissection took place, when the embryos had fewer than 50 cells (Fig. S7A,B, left; Table S3) and lasted until later developmental stages (Fig. S7A,B, right; Table S3), at which the altered blade topology was particularly obvious, especially when microdissection took place at E_0_ and E_1_. Therefore, removal of the stalk before the 4-cell stage resulted in a long-term alteration of the spatial arrangement of cells within the monolayer lamina.

In summary, in embryos from which the maternal stalk was severed before the 8-cell stage, nearly all cells maintain their l/w ratio, but these cells were less rectangular and smaller than in intact embryos. This change in cell shape may contribute to an altered cell arrangement within the embryo lamina, together with the rounder shape of the embryo itself, which, *de facto*, reduces the relative abundance of cells at the periphery of the lamina and, hence, of cells with only three neighbours.

### The disruption of the body planes is due to early longitudinal cell divisions in Phase I

So far, we have shown that the separation of the E/Z/E from the maternal stalk results in morphological defects in PhII embryos. Namely, the embryos developed as disc-like blades, with smaller, irregularly shaped cells, which are haphazardly arranged within the lamina. To determine whether MUM controls cell shape and size as the main targets, thereby affecting embryo shape, or whether MUM controls embryo shape, which in turn affects cell shape and size, we looked at the earlier steps of embryo development. We observed that severing the embryos from the maternal stalk modified the orientation of their initial cell divisions. In contrast to intact embryos (Fig. 4B, left), microdissected embryos initiated longitudinal cell division as early as the 2-cell stage (Fig. 4B, right). In fact, longitudinal cell division parallel to the position of the stalk took place immediately after the separation of the E/Z/E from the maternal tissue (Fig. 4B, right), whereas in intact embryos, longitudinal cell division rarely took place before the 8-cell stage (Fig. 4B, left). This difference in the timing of longitudinal cell division suggests that MUM controls the orientation of the cell divisions in the very initial stages of embryogenesis. Nevertheless and interestingly, even in E_0_ and E_1_, the first cell division is always transverse.

This result makes it possible to identify the cause of the disorganisation of the embryo morphology observed in phase II. In early phase I, the primary longitudinal, apico-basal axis is not yet fully established, and the occurrence of longitudinal divisions parallel to this axis in the absence of MUM results in embryo growth along the medio-lateral axis. This contrasts with intact embryos exposed to MUM, where a linear stack of eight cells is produced before the second body axis is established.

It is interesting to note that, in the absence of MUM, only divisions parallel or perpendicular to the stalk (before dissection), and no oblique orientation, were observed in the growth steps following stalk removal (Fig. 4B). Therefore, MUM does not control the overall rate of cell division (Fig. 4A) or the orientation of cell division per se, but only the conditions of when and where longitudinal divisions occur.

### MUM is a short-range signal that crosses a dead cell

#### MUM is a local signal

**Fig. 5.**
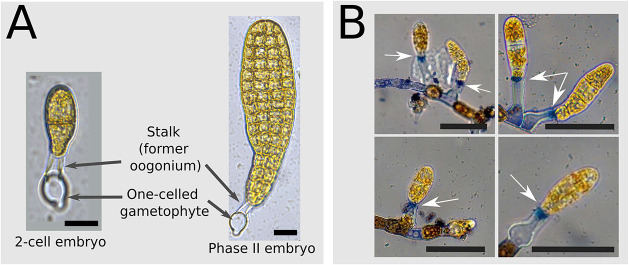
**Transport of MUM from the female gametophyte to and through the embryo*.*** (A) The embryo does not require a developed maternal tissue. Two-cell stage and phase II embryos attached to a one-celled gametophyte are shown. (B) Trypan Blue staining of the stalk. Embryos are retained on the stalk (white arrows). Tissue exposed to Trypan Blue showed blue precipitates in the stalk. Staining was stronger at the apex of the stalk than at its base. Although some empty cells of the maternal gametophyte were also stained, indicating that they are dead (top), live cells remained brown (early-stage embryos and gametophyte cells). Four examples are shown. Scale bars: 50 µm.

In nature, normal embryos can grow from a maternal gametophyte made up of only one cell. Similarly, in the lab, meiospores collected from a wild fertile sporophyte that are immediately exposed to white light germinate and grow into one cell and immediately produce an oogonium (Lüning, 1981; Theodorou et al., 2021). Embryos growing from the fertilised egg extruded from this single cell develop similarly to embryos growing from large filamentous female gametophytes (Fig. 5A). This feature has also been reported in other *Saccharina* species (Kanda, 1936). Therefore, MUM can act locally at the level of the stalk or of the gametophyte cell with which it is in contact.

By immersing severed E/Z/E in Petri dishes containing intact fertile gametophytes, we tested whether MUM diffuses in seawater. Microdissected embryos growing in the same seawater as intact gametophytes did not develop differently from those isolated in a distinct Petri dish: both groups grew with developmental defaults. This result suggests that there is no molecular or chemical compound excreted from the maternal gametophyte that diffuses in seawater and controls the development of the embryo.

These two experiments support the hypothesis that MUM is neither a signalling factor necessitating transport from cell to cell in the female gametophyte to the stalk, nor a signalling factor diffusing in seawater to the E/Z/E. Therefore, MUM is a signalling factor related to the stalk itself.

#### The stalk is a dead structure

The literature indicates that, in Laminariales, the oocyte is extruded from the mature oogonium, whereby the oogonium ejects its cellular content from an opening at its apex, thereby producing a protoplast (the egg) on the one hand, and the remnant, inert cell wall of the oogonium on the other hand (Lüning, 1981; Klochkova et al., 2019). We confirmed that the remnant oogonium cell wall in *S. latissima* is a dead structure by staining it with Trypan Blue (TB), a negatively charged dye, which is not taken up by living cells with intact membranes (Farah et al., 2015). TB accumulated in the stalks as well as in dead gametophyte cells that were used here as controls (Fig. 5B, top-left panel). Interestingly, TB staining was stronger at the apex of the stalk near the connection to the embryo. Previous experiments have shown that the stalk interior is highly viscous, and we have observed viscous material leaking from the stalk when pierced upon mechanical or laser manipulation (Boscq et al., 2022). Therefore, in addition to confirming that the stalk is a dead structure, TB staining showed that its interior is dense and heterogeneous.

## DISCUSSION

### The control of longitudinal cell division during the embryogenesis of Laminariales is a labile trait

At the morphological level, the first body axis of the embryos of Laminariales is the longitudinal axis (defined here as being the *x*-axis; Theodorou and Charrier, 2023), reportedly established upon elongation of the zygote after fertilisation of the spherical egg (Kanda, 1936). The elongated zygote then divides transversally. Interestingly, different genera in order Laminariales display different distributions of transverse and longitudinal cell division orientations (Kanda, 1936, 1938, 1941; Sauvageau, 1918), resulting in embryos with varied l/w ratios. Therefore, control of the secondary axis (denoted as Y) establishment is a common but plastic trait among individual embryos and among Laminariales species. Patterns of cell division orientations are also dramatically modified when eggs have detached naturally from the stalk (Kanda, 1936; see Sauvageau, 1918 for *Laminaria flexicaulis*, the former name of *Laminaria digitata*, which also belongs to order Laminariales). In *S. latissim*a, detached eggs display abnormal cell division and stop growing soon after fertilisation, which results in over 60% of the embryos not developing beyond the 2- to 3-cell stage (Klochkova et al., 2019). Removing flagella leads to the same outcome. Our results show that the control of longitudinal cell division can also occur after fertilisation and when embryos attached firmly to the stalk are mechanically separated from the maternal tissue by severing the stalk.

### Body planes and tissue patterning of the *Saccharina* embryo are controlled by a maternal unknown message (MUM)

**Fig. 6.**
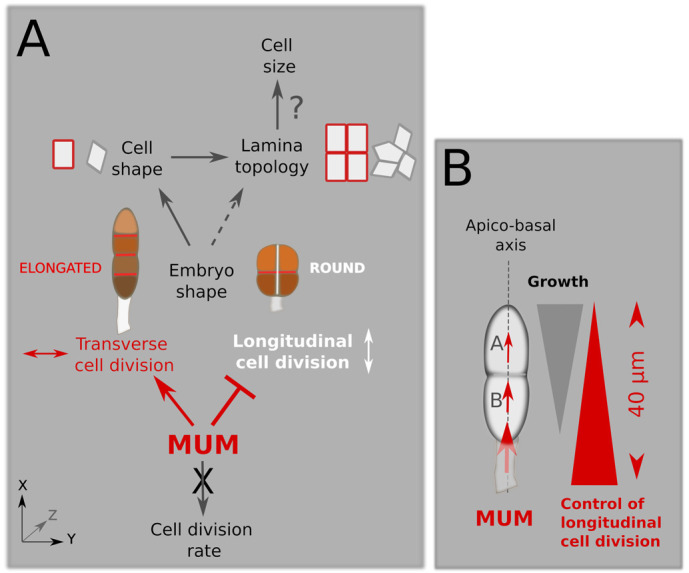
**Range of action of maternal unknown message (MUM)*.*** (A) Model of the control on embryo body planes, tissue topology and cell shape by MUM. MUM negatively controls longitudinal cell divisions (white), thereby promoting transverse cell divisions (red). Elongated embryos are formed of cuboid cells (top, red-outlined rectangles) arranged in rows and columns (left-hand side, stack of brown cuboids in phase I embryos; top, right-hand side, tissue topology made of aligned red-outlined rectangles in phase II embryos). In the absence of MUM (e.g. amputation of the maternal stalk), the embryo is rounder and smaller (right-hand side). Cell shape and topology of the lamina are altered (top, light grey-outlined parallelepiped), resulting in the reduction in cell size. (Bottom) Cell division rate is not under the control of MUM. (B) Acropetal diffusion of MUM. MUM is a signal diffusing from the stalk, through the basal cell (B) to the apical cell (A) of the embryos. Grey arrowhead indicates basipetal gradient of growth activity (Boscq et al., 2024). Red arrowhead indicates a cropetal gradient of inhibition of longitudinal cell division. The range of action of MUM is about 40 µm. Beyond this distance, once the embryo is approximately 8 cells long, the cells divide longitudinally.

We showed that separating the embryo from the maternal tissue by cutting the stalk in the very early stages of embryogenesis resulted in several embryogenesis defects. The most conspicuous defect was the alteration of the body axes. Where intact embryos grew in length, embryos separated from the maternal tissue tended to grow as a disc, thereby losing their anisotropic shape. Examining the very first steps of embryogenesis, we noticed that longitudinal cell divisions occurred earlier than in intact embryos. Intact embryos first divided only transversally up to the 8- to 10-cell stage, whereas the microdissected embryos initiated longitudinal cell division as soon as the 2-cell stage. Noteworthily, in the latter case, longitudinal cell division never occurred before the two-cell stage, suggesting that the egg axis is aligned with the stalk before that stage, perhaps before the oocyte is extruded. Therefore, although the *x*-axis of the zygote seems to be established at the egg stage at the latest, its maintenance and the establishment of the *y*-axis depends on the contact of the sporophyte with the female gametophyte. Therefore, we introduced the idea of MUM, a maternal message of yet unknown nature, which controls the body planes in the *S. latissima* embryo (Fig. 6A).

Embryos growing with a sectioned stalk had also malformed and smaller cells, as well as a disorderly arrangement of cells within the embryo lamina. They may each result from the alteration of embryo shape, because rounder laminae are less prone to arrange cells in rows and columns than elongated embryos. In turn, altering the topology may force the cells to grow less in size while maintaining the same rate of division, this latter parameter not being under the control of MUM (Fig. 6A). As a result, severed embryos are smaller.

### MUM is a local signal emitted from the stalk, acting at the very beginning of embryogenesis

MUM does not appear to be a signalling factor that diffuses in seawater from fertile female gametophytes, but instead requires direct contact with the E/Z/E through the stalk. In addition, MUM action does not require a developed maternal environment, because a one-celled gametophyte can bear normal embryos. This suggests that MUM acts locally at the level of the stalk.

Female centrioles, through their role in the formation of flagella anchored to the stalk, are thought to be responsible for the formation of the embryo's longitudinal axis (Klochkova et al., 2019). When the egg is devoid of flagella (e.g. manually pulled away from the stalk, or separated by shaking the flask), most eggs stop growing (Klochkova et al., 2019); however, the microdissection method that we used usually left some of the stalk attached to the E/Z/E and, therefore, the flagella were most likely not sectioned, but only damaged. Furthermore, the female centrioles, which are necessary for flagella, degrade as early as the zygote stage (Motomura, 1990, 1991), but the morphologies reported here were observed when the stalk was severed up to the 4-cell stage (even the 8-cell stage for some parameters). Therefore, the persistence of MUM action throughout most of phase I embryogenesis precludes the possibility that MUM is the flagellar structure itself. The same reasoning applies to male centrioles, which replace female centrioles at the zygote stage (Motomura et al., 2010) and therefore were already present and correctly positioned within the cells when most microdissection experiments took place.

The region of the stalk interacting with the E/Z/E and where the flagella emerge coincides with the presence of a thick cell wall collar (Kanda, 1936; Klimova and Klochkova, 2017), which may clasp the basal region of the zygote and subsequently the early embryo, thereby preventing its growth in the *y*-axis. In control embryos, inhibition of longitudinal division occurs up to the 8-cell stage, when the embryo is 50-60 µm long. It is therefore difficult to imagine that a mechanical constraint exerted by a constricting collar at the top of the stalk acts beyond the base of the zygote/embryo itself. In the embryo of *Eckloniopsis radicosa* (Kjellman) Okamura (Laminariales, Lessoniaceae), the basal cell grows within the stalk and it does not divide longitudinally, while the rest of the embryo outside the stalk actively divides longitudinally, and, as a result, grows as a fan-shaped embryo (Kanda, 1941). Therefore, in this species of Laminariales, the constricting collar does not seem, as expected, to inhibit longitudinal cell divisions beyond the cell in direct contact with it.

The polysaccharides present in the stalk or at the stalk-embryo junction could be good candidates for being MUM. In the kelps *Saccharina* and *Alaria,* a cocktail of α-D-glucose, α-D-mannose and L-fucose accumulate specifically at the junction between the stalk and the egg (Klimova and Klochkova, 2017; Klochkova et al., 2019). This cocktail is no longer detected when the egg is free floating, unattached to the stalk or to another substratum. The role of sugars as signalling molecules involved in development remains to be demonstrated in brown algae, but is well known in other organisms such as plants (Mishra et al., 2022; Wang et al., 2021). Therefore, this cocktail of polysaccharides located at the stalk-embryo junction may participate in the signalling from the intact stalk – a condition that would be required to maintain the proper cocktail – to the embryo. Mucosal compounds trapped in the stalk (Klochkova et al., 2019 and our own observations) may also be another source of glycoconjugates acting as MUM.

### Could the cell wall present in the flanks of the remaining pieces of the stalk be MUM?

We have shown that embryos with pieces of cell wall remaining from the severed stalk have altered morphologies; it is therefore highly unlikely that these pieces are sufficient to carry MUM. Interestingly, in other families of Laminariales, the basal cell of the embryo (e.g. in *Eisenia bicyclis, Ecklonia* sp*.*) or the maternal gametophyte itself (in *Undaria* sp*.*) grows into the stalk after the egg has been extruded (Dries et al., 2024; Kanda, 1941). During growth, the contact of this gametophyte cell or, in particular, the basal embryo cell with the inner cell wall of the stalk, does not modify their fates (Kanda, 1941). Therefore, unlike the cell wall of the rhizoid and thallus cells of the *Fucus* brown alga embryo (Berger et al., 1994; Bouget et al., 1998), the cell wall of the stalk of these Laminariales does not seem to carry cell fate determinants. This is even less likely in *Saccharina*, where the embryo is not in direct contact with the lateral walls of the stalk, but only with its collar.

Furthermore, we have recently shown that the basal cell of the early stage *Saccharina* embryo is necessary for the negative control of longitudinal cell divisions (Boscq et al., 2024). This result, together with those of the microdissection of the stalk in the present study, strongly suggest that MUM diffuses from the stalk into the basal cell of the embryo and, then, further upwards to the apical cell of early embryos up to the 8-cell stage (Fig. 6B). Because the first longitudinal division usually occurs in the upper half of the embryo, the first cells out of MUM control are therefore apical cells. This characteristic is shared by many members of Laminariales (Sauvageau, 1918) and even of other orders (e.g. *Sacchoriza* and Tilopteridales; Fritsch, 1945; Norton, 1972). This indicates a ubiquitous and acropetal mode of action of MUM, in which the inhibitory effect is strong in the basal part of the embryo, but diminishes towards the apex. In our study, the first longitudinal cell divisions occur at least 40 µm away from the stalk, which would be within the range of the actions of MUM. Beyond this distance, cells appear to begin to divide longitudinally (Fig. 6B).

This contrasts with the recent findings of Dries et al. (2024) on kelp *Undaria* (Alariaceae, Laminariales), where the walls of a severed stalk are thought to carry a locally active signal. Whether the 30 million years separating the evolutionary trajectories of *Saccharina* and *Undaria* (Starko et al., 2019) or the different growth rates and cell division patterns of their respective embryos (Dries et al., 2024) can account for these different mechanisms remains a possibility.

### How could MUM diffuse acropetally from the intact stalk to the apical cell of the embryo?

A distance-based inhibitory relationship with a signalling molecule is not a foreign concept in brown algae, especially kelps. For example, the reproductive structures (sori) are formed away from the growing region that surrounds the basal transition zone, due to the action of a sporogenesis inhibitor (Buchholz and Lüning, 1999; Pang and Lüning, 2004), which may be auxin (Kai et al., 2006). Plasmodesmata, which have been observed in phase II *Saccharina* embryogenesis (Theodorou and Charrier, 2023) can, if also present earlier (preliminary results seem to indicate so), contribute to the formation of a symplastically diffusible gradient of molecules originating from the stalk and forcing transverse cell divisions throughout the embryo. This action could be achieved by any type of compound, as long as its size does not exceed 20-40 kDa (10-20 nm) (Nagasato et al., 2017), which is small compared with land plant plasmodesmata, but large enough to allow signalling molecules such as auxin to pass through.

Furthermore, for most morphometric and topological parameters observed in this study, the effect of MUM was stronger at the egg and zygote stages than at the 4-cell and 8-cell stages, and in early phase II embryos. The embryo response was gradual from the egg stage to the 8-cell stage. By the time the embryo reached phase II, separation from the maternal stalk no longer had any effect on the morphological parameters that we observed, at least up to the 300-cell stage. Therefore, the effect of MUM on embryogenesis seems to occur primarily during the very first steps. However, in embryos that have grown up to 1000 cells (end of phase II), the base of the blade remains the narrowest part of the embryo, reflecting the maintenance of a low rate of longitudinal cell divisions specifically in this area (Theodorou and Charrier, 2023). Because the embryo remains attached to the female gametophyte up to that stage, it is possible that MUM still acts very locally and, to a lesser extent, in the basal region of the embryo up to that stage, where it reinforces the embryo apico-basal polarity established in the earlier phases. Thus, a range of action of MUM of at least 40 µm would last longer than the duration of phase I, i.e. beyond the 8-cell stage. Alternatively, another factor could relay the inhibitory effect of MUM on the widening of the base after the 8-cell stage. In conclusion, a chemical basis remains the most likely explanation for the acropetal diffusion of MUM in the embryo and its inhibitory effect on longitudinal divisions over a distance of around 50 µm.

### Evolution of the maternal tissue-embryo connection

The ancestor of the Stramenopiles, the main group of the brown algae, diverged from their eukaryote ancestors at least 1 billion years ago (Burki et al., 2020). Among brown algae, at least four orders (Laminariales, Sporochnales, Desmarestiales and, to a lesser extent, Tilopteridales) display a stalk-mediated, physical connection of the embryo with the maternal tissue that persists over the egg or zygote stages (Fritsch, 1945). In Fucales, although stalks connecting eggs with maternal tissue are common (Burridge et al., 1993), this connection is transient and does not persist after fertilisation.

Compared with other multicellular organisms, this type of interaction is rare in the tree of life. In the green alga *Coleochaete* sp. (Chlorophyta), the zygote remains attached to the female gametophyte until it completes a series of divisions leading to the release of up to 32 biflagellate spores. This release of spores rules out any physical involvement of parental tissues in the development of the haploid embryos. Furthermore, this is a case of matotrophy only (Haig, 2015), unlike the case of *Saccharina*. In several red algae (Gelidiales and Gracilariales), the (carpo)sporophyte develops on the maternal gametophyte, but in the form of gonimoblasts, which are diploid filaments that produce carpospores, which disperse before developing into diploid (tetra)sporophyte embryos away from the maternal tissue (van der Meer, 1979). Thus, maternal tissue does not directly control embryogenesis, and its relationship with the carposporophyte (filamentous gonimoblast) is trophic (matotrophy). In the red alga *Palmaria palmata*, however, the situation is similar to that of *Saccharina*: the embryo develops as a macroscopic elongated blade, in physical contact with dwarf haploid maternal tissue (Gall et al., 2004; van der Meer and Todd, 1980). However, it is not known whether the latter has an impact on the development of the embryo's growth axes.

In contrast, in bryophytes (group comprising the mosses, liverworts and hornworts), the developing embryo is surrounded by a layer of maternal cells, making up the archegonium (Naf, 1962) and subsequently covered by the calyptra, which is a cell layer of female origin protecting the embryo from desiccation (Budke et al., 2012). Subsequent evolution of this branch led to increasingly protected embryos surrounded by additional layers of maternal cells, as in the seeds of land plants, where the embryo is embedded in endosperm, a triploid tissue resulting from the fertilisation of a diploid maternal tissue, and the integuments and fruit differentiating from maternal tissues. Requirement of both the integuments and the endosperm for the proper developmental pattern of the embryo has been demonstrated (Kunieda et al., 2013; Weijers et al., 2003). A very complex process has been uncovered, whereby auxin first produced by the endosperm controls the differentiation of the integuments, which then become a source of auxin for the development of the embryo (Figueiredo and Köhler, 2016; Figueiredo et al., 2016; Robert et al., 2018).

That Laminariales, Tilopteridales, Sporochnales and especially Desmarestiales, which diverged early (Bringloe et al., 2020; Silberfeld et al., 2010), are pioneers of a parental-embryo physical connection within the brown algae is congruent with the fact that they diverged independently during the radiation, giving rise to most current brown algal orders. In any case, this particular mode of maternal-embryo interaction has clearly enabled Laminariales to become the largest and most morphologically complex brown algae to thrive in the oceans.

## MATERIALS AND METHODS

### Algal culture

The culture and production of embryos of *S. latissima* (Arthrothamnaaeae, Laminariales and Phaeophyceae) were carried out according to Theodorou et al. (2021). Female (F1) and male (M1) gametophytes with a fixed genotype were used to produce all the embryos. These genotypes were selected from the offspring of one mature sporophyte collected on the beach at Perharidy (Roscoff, Brittany, France) (48°43'33.5″N, 4°00'16.7″W) based on their growth rate and sexual compatibility when cultured *in vitro*. F1 and M1 gametophytes were produced by vegetative multiplication as described by Theodorou et al. (2021). Gametes were obtained from the maturation of gametophytes under 16 μmol photons m^−2^·s^−1^ white light intensity and 14:10 light:dark photoperiod at 13°C. Embryos were observed after transferring the cultures to higher light intensity (50 μmol photons m^−2^·s^−1^) for 1 week.

### Excision of the maternal tissue at different developmental stages

The separation of embryos from their maternal stalk was carried out by using pulled glass micro-needles. First, glass micro-needles were prepared by pulling glass capillary tubes (GC100F-10) with a pipette puller (SU-P97 Flaming/Brown type micropipette puller) using the following programme: heat, 564°C; pull, 70 U; velocity, 70 ms; time, 250 ms. After pulling, the tip of the needle was sharpened to ensure precision cutting. Second, developing, intact eggs, zygotes and embryos (E/Z/E thereafter) were selected under a flow hood using an inverted microscope (Olympus CKX41 Inverted with phase contrast) and the tip of the stalk was cut using the glass needle in a cutting motion, while holding the embryo down on the bottom of the dish. The microdissected E/Z/E was transferred into a distinct new Petri dish and filled with filtered natural seawater (NSW), using a non-pulled capillary and a manual microinjector (Eppendorf CellTram Air 5176). This action was repeated *n*=100 for eggs, *n*=100 for zygotes, *n*=80 for 2-cell, *n*=80 for 4-cell and *n*=40 for 8-cell embryos, as well as *n*=40 early phase II embryos (corresponding to 8-cell to ∼1000-cell embryos, see Theodorou and Charrier, 2023 for the definition of the embryogenesis stages). In our experiment, the occurrence of embryos naturally detached from the maternal gametophytes was excluded, because we manually transferred the sectioned E/Z/E into a new Petri dish.

Furthermore, from the frequency of observed embryos with altered morphology in a population of intact embryos (Table S2), we estimated, using the binomial law, the probability of having only morphologically normal embryos developing attached to F1 female gametophytes grown in the presence of the M1 male gametophytes, and the expected number of abnormal embryos per sample, per group of microdissected embryos. Details on the calculation using the binomial law are given in the supplementary Materials and Methods.

### Cell staining

#### Trypan Blue

A drop of Trypan Blue (TB) was added to fertile female gametophytes of *S. latissima* immersed in ∼0.5 ml of NSW, followed by observation in bright field microscopy (DMI8, Leica Microsystems).

#### Calcofluor white

Staining of cell walls from embryos separated from the maternal tissue was performed at different times post excision. Embryos were fixed for 1 h in equal parts of 4% PFA in H_2_O and NSW. After fixation, the samples were washed in NSW and twice in PBS to remove any excess fixative. Subsequently, they were incubated with 20 µM Calcofluor White (Sigma-Aldrich) for 3 days at 4°C in the dark. After incubation, the samples were washed three times with PBS to remove any unbound dye. Finally, the algal samples were mounted using Cityfluor mounting medium (Electron Microscopy Sciences).

### Image acquisition

All experiments of time-lapse microscopy of growing embryos were recorded under a bright-field microscope (Leica DMI600 B, Olympus CKX41 or Leica DMi8 inverted phase contrast microscopes) equipped with a DFC450C camera with acquisition intervals of 2 to 24 h for a duration of at least 10 days after excision. The required temperature and light were set in a carbonate-glass chamber fitted to the microscope and equipped with a thermostatically controlled airflow system (Cube and Box, Life Imaging services) and commercially available LED white light sources. Observations of cell wall staining (see the experimental procedure for Calcofluor White staining) were performed with a Leica TCS SP5 AOBS inverted confocal microscope (20× objective/N.A. 0.70 and correction 0; Exc/Em band wavelengths: 405/561-596 nm; pinhole, 60.6 µm).

### Manual segmentation

Automatic segmentation proved to be challenging due to the constant pigmentation changes of the cells transitioning from high to low colouration. Additionally, daily exposure to UV light required to visualise Calcofluor White staining proved to be detrimental to the algae. Therefore, manual segmentation was carried out on bright-field images. To minimise image deformation, flat-growing embryos were preferentially chosen. *Z*-stack images of time-lapse acquisition were segmented manually by the same person, using Fiji (ImageJ2 version 2.9.0) (Schindelin et al., 2012) and the outlines were implemented in Inkscape (version 1.2). Resulting cell wall contours were analysed using in-house software (see below). The programme extracted multiple quantitative parameters for each embryo lamina (blade) and its cells.

### Quantitative morphometry

Cell wall vector graphics were processed in dedicated software written in object-oriented python 3 (Van Rossum and Drake, 2011) that we called blade_painter (available at https://gitbio.ens-lyon.fr/igfl/charrier/blade_painter). Reading the svg file, blade_painter extracts various geometric properties for cells and laminae (Rosin, 2005), namely (1) each cell contour, from which were directly derived the perimeter length and surface area; (2) convex hull, used to compute the minimal bounding rectangle [MBR; the main axis and length/width (l/w) ratio (or elongation) of the cell were assumed to be those of the MBR]; (3) rectangularity, computed as the proportion of overlapping surface between the cell and its MBR, rescaled to the same area; (4) neighbouring cell counts, where two cells were considered neighbours if they shared at least 200 nm of cell wall (this threshold can be modified as a software parameter); (5) blade area; (6) main and secondary axes of orientation and length of the blade, computed according to Fletcher et al. (2013).

### Statistical analysis

Data collected from the segmented cell and blade contours were analysed using standard python3 libraries, namely pandas (The Pandas Development Team, 2020) and scipy (Virtanen et al., 2020). All pairwise comparisons for cell data were conducted using the Student's mean comparison test with Welch's correction, except for neighbouring cell counts and orientation, which were compared using a χ² test. Blade data were compared using the nonparametric Mann–Whitney test. All the tests are two-tailed.

### Growth rate

For each observed blade, the date of observation was set to *t*=0 at the transition from phase I to phase II (first longitudinal division). Linear regression was performed on the logarithm of cell number as a function of time, for −48≤*t* ≤72. The increase rate was computed as *r* in the equation *N*=*N*_0_×*r*^*t*^, thus derived from the slope of the regression line *log*(*N*)=*log*(*N*_0_)+*log*(*r*)×*t*. From *r*, we inferred the doubling time *τ*=1/*log*_2_(*r*).

## Supplementary Material



10.1242/develop.202732_sup1 Supplementary information

Table S1. (S1A) (Left) Counts and (Right) percent of embryos i) growing normally, ii) with a lower growth rate, and iii) with morphological alteration, for stages at which microdissection was performed. The percentage of morphologically abnormal embryos observed in the control (intact) population, due to the morphological plasticity of the genetic strains, is highlighted in blue. (S1B) Probability, calculated from the binomial law, of frequency of embryos with abnormal morphology in the segmented samples (based from observation in populations of intact embryos, Table S1A). From this probability and the size of each sample, the expected number of abnormal embryos is calculated. See Experimental procedures for the calculation of these probabilities.

Table S2. Morphometric parameters of the embryos (blades). The table is sub-divided into 3 sections based on the size of the observed embryos (expressed in the number of cells nCells, 1st and 4th columns). “In” are intact embryos. Not all embryos have data available for all three size ranges.

Table S3. Cell morphometrics in each embryo (blade). Time point and number of cells are indicated for each embryo. Each cell (one row) was studied for different morphometric parameters. Those relevant for this study are displayed. The data are shown for the three ranges of embryo size (in cell number; e.g. [20:47] cells, indicated by the first column).

Table S4. P-values of statistical tests comparing morphometric parameters characterising *Saccharina* embryos and their cells. *t*-tests were used to compare the cell features, Mann-Whitney tests to compare embryo blades and X^2^ for the analyses of the number of cell neighbours. The quantitative value of each parameter and for each developmental stage at which the stalk was severed (E_*n*_ and PhII), is presented as the function of the time window (expressed as the number of cells). In green, p-values lower than 5.10^-2^. In the main figures presented in the article, only p-values resulting from the comparison between the microdissected embryos and the intact controls are shown.
