# Supplementary material for: MUM, a maternal unknown message, inhibits early establishment of the medio-lateral axis in the embryo of the kelp Saccharina latissima
Source: Development. 2024 Sep 13;151(20):dev202732. doi: 10.1242/dev.202732 (PMC11423915; doi:10.1242/dev.202732)
Supplement: Supplementary information [file develop-151-202732-s1.pdf]

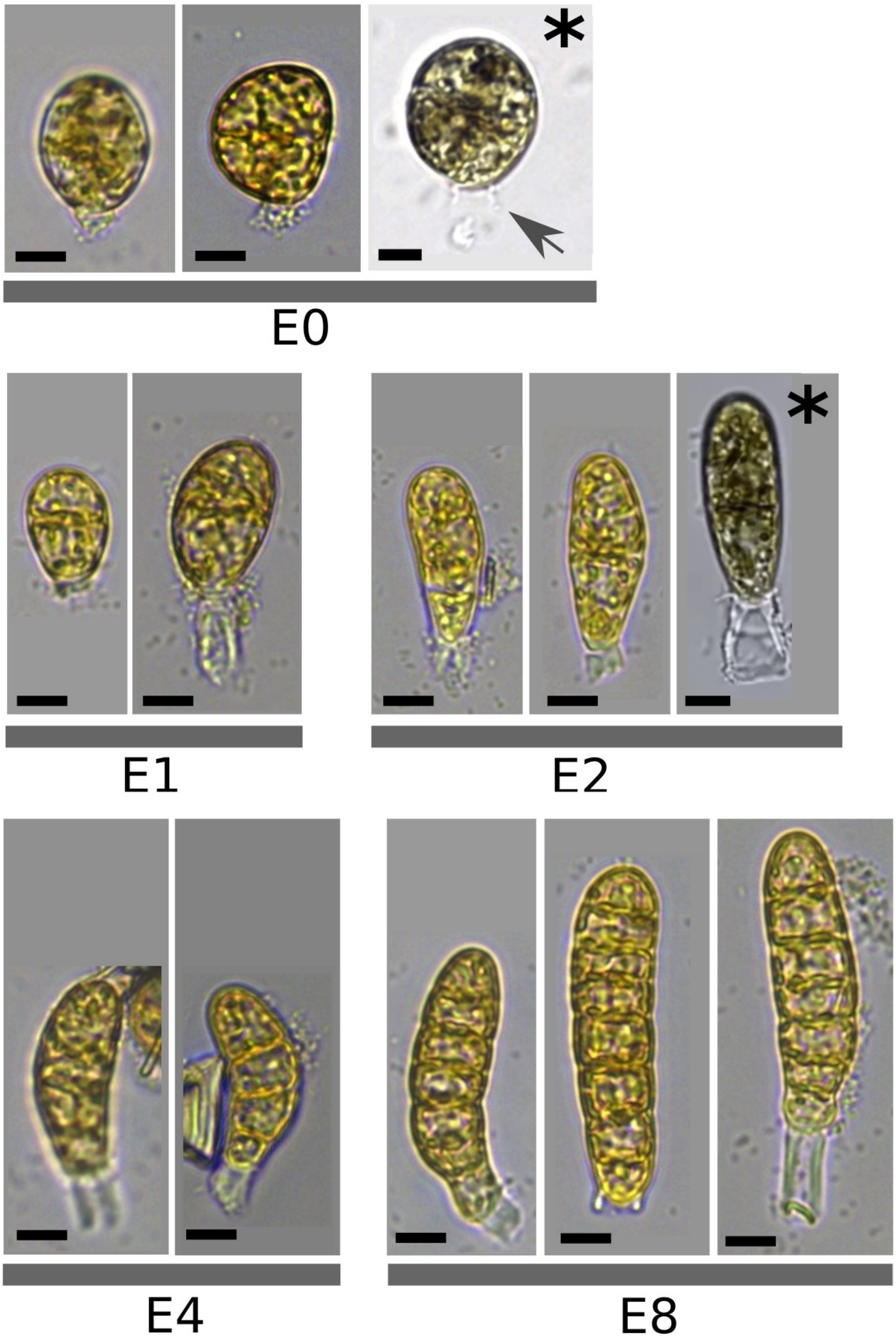

Fig. S1. Microdissected embryos showing the remnants of the cell wall of the stalk. Embryos microdissected from the egg stage (E_0) up to the 8-cell stage (E_8) were photographed one day after microdissection. Note the remnants of the cell wall of the microdissected stalk in the basal region of the embryos, which vary in size, depending on where the stalk was severed and how clean the cut was. Movie 1 and movie 2 show the time-lapse monitoring of the two embryos with an asterisk. Scale bar = 10 µm.

Development • Supplementary information

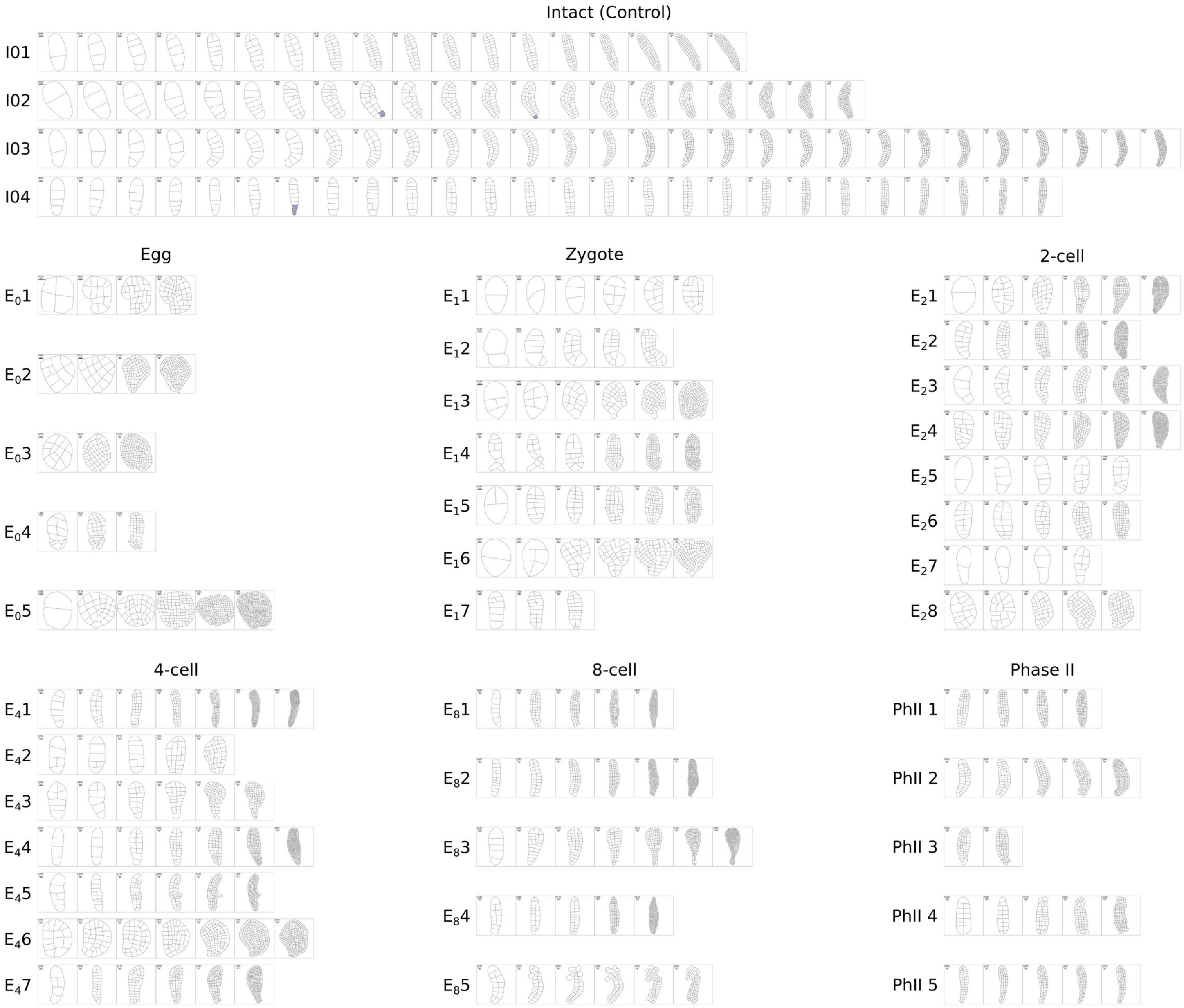

Fig. S2. Results of manual segmentation of the embryos

The cell outlines were drawn manually from a series of bright-field z-stack images (Stack focuser, see Materials and Methods section) and used for quantitative analyses of the morphometry parameters. The stages at which the stalk was severed is indicated with the code used in the article (e.g. E_0 for the separation from the stalk at the egg stage). I is for intact embryos. Replicates are indicated with number n (e.g. E_8n).

Development • Supplementary information

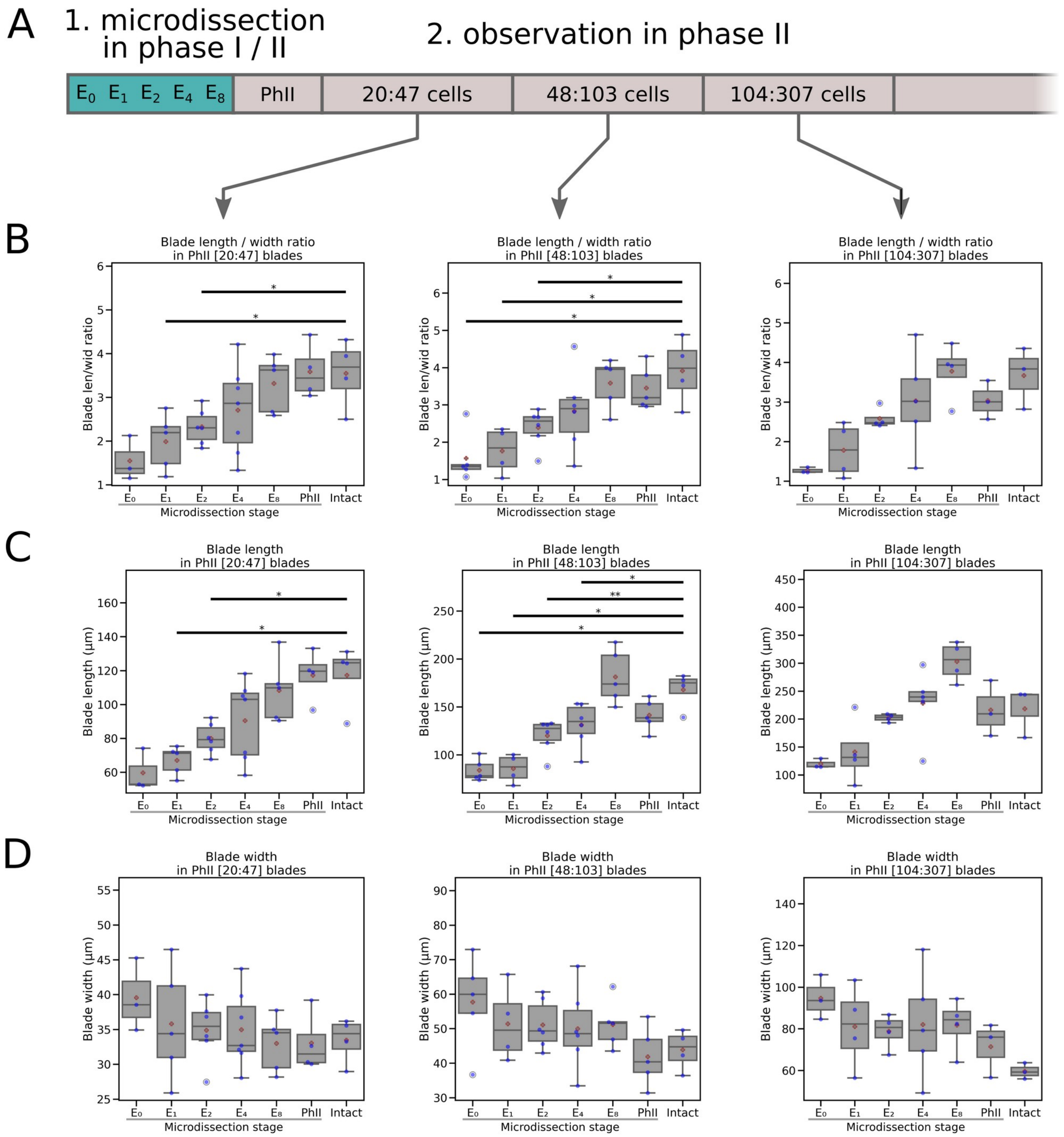

Fig. S3. Impact of severing the embryo from the maternal stalk on blade shape

(A) Experimental design. Microdissection was performed at the E_0, E_1, E_2, E_4, E_8 or PhII stage, then the embryo morphology was analysed in Phase II, in three intervals of number of cells: 20-47, 48-103 and 104-307. Control (intact) embryos were observed at the same stage. (B-D) Distribution of the blade length / width ratio (B), blade length (C) and blade width (D) was represented as box plots depending on the stage of microdissection. Box plots are shown for three intervals of Phase II growth: for embryos of 20-47 cells (left), 48-103 cells (centre) and 104-307 (right) cells. Within the box, the thick red-outlined plus sign shows the mean; the middle line is the median (2nd quartile);

Development • Supplementary information

the box includes values between the 1st (25%) and 3rd quartiles (75%); the whiskers show the extent of observations around these values, up to 1.5 times the difference between the 1st and 3rd quartiles; other observations are shown as outliers (circles). P-values of the Wilcoxon test are indicated as follows: * $p<5.10^{-2}$; ** $p<1.10^{-2}$; *** $p< 10^{-3}$. Results in late embryos of [104:307] cells were not supported statistically because the sample size was small: 26 embryos *vs* 34 and 35 in [20:47] and [48:103] cell embryos, respectively.

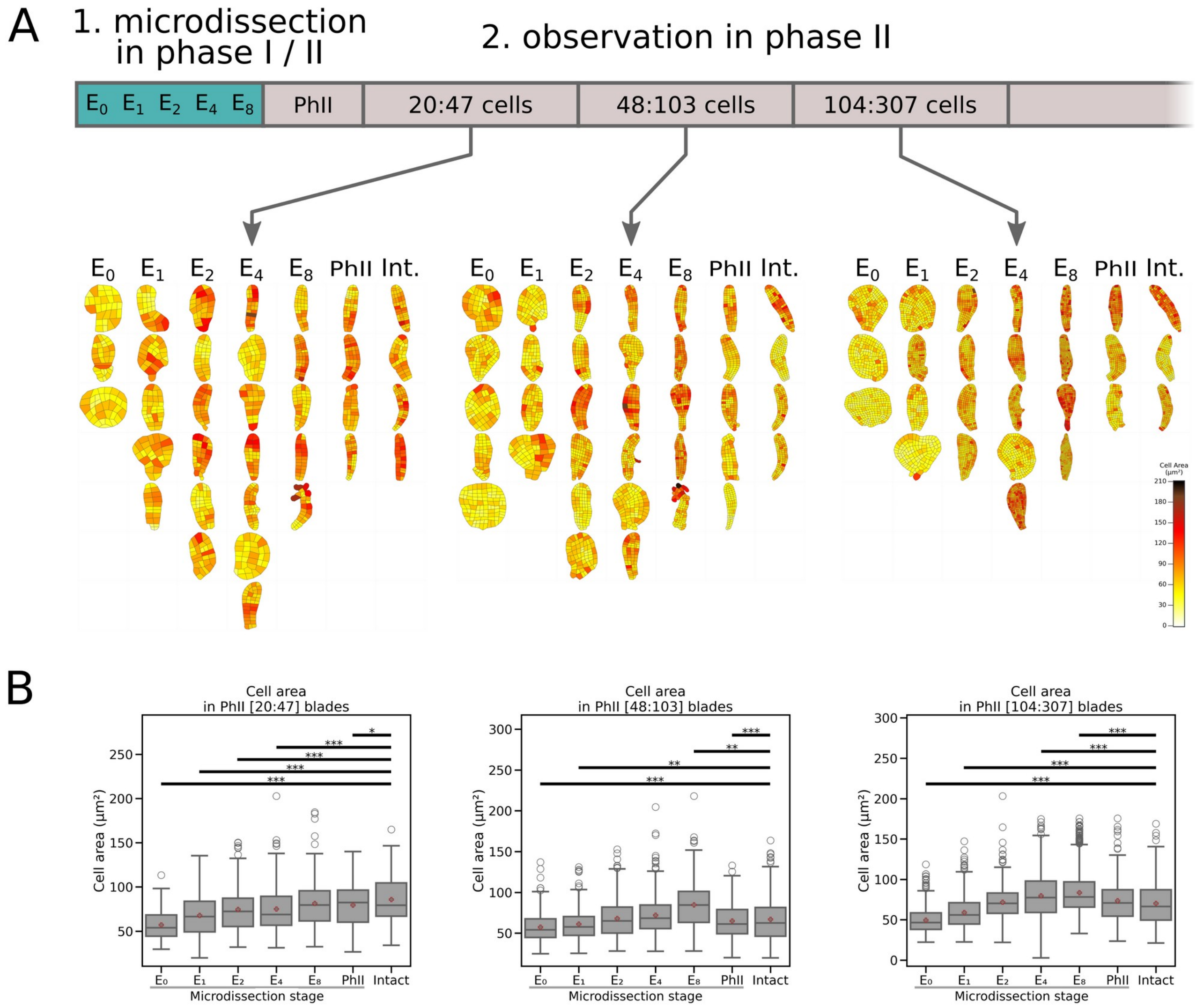

Fig. S4. Impact of severing the embryo from the maternal stalk on cell surface area.

(A) Experimental design (see Fig. S3 for details) and heatmap for the area coloured on segmented embryos at different developmental stages (20–47-cell, 48–103 and 104–307-cell embryos, respectively left, centred and right panels) grown after microdissection of the stalk at E_0, E_1, E_2, E_4, E_8 and PhII stages. Colour scale is indicated on the bottom right-hand side of the figure. The scale was adjusted, with the image of each embryo (blade) occupying roughly the same space regardless

of cell number. (B) Box-plot representation of cell area. The thick red-outlined plus sign in the middle of the box shows the mean; the middle line is the median (2nd quartile); the box includes values between the 1st (25%) and 3rd quartiles (75%); the whiskers show the extend of observations around these values, up to 1.5 times the difference between the 1st and 3rd quartiles; other observations are shown as outliers (open circles). Results of the *t*-test are indicated by p-values: *$<5.10^{-2}$; ** $<1.10^{-2}$; *** $< 10^{-3}$.

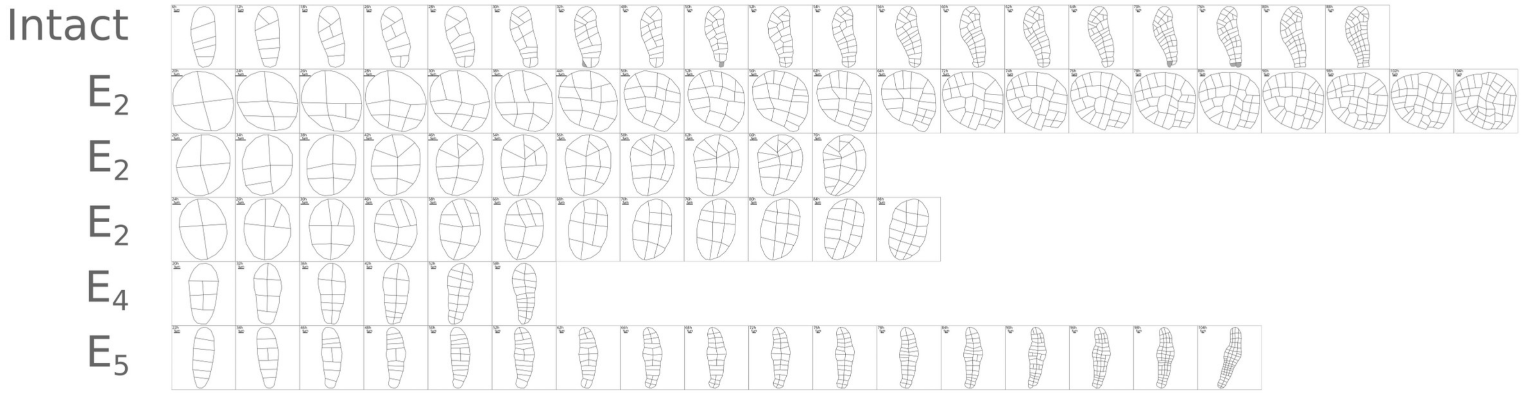

Fig. S5. Time series of microdissected embryos.

Photos of embryos imaged every 2 h were segmented at each additional cell division (the time interval between cell division is about 24 h, so that not all the images were segmented). Each line represents one embryo, the stage at which microdissection took place is indicated in the left margin.

E_5 is a 5-cell stage embryo. Scale bar is indicated for each embryo. Note that the scale differs mainly between intact and microdissected embryos, because the scale was adjusted so that the boxes containing the image of each embryo (blade) are all the same size.

m the 1st (25%) to the 3rd quartiles (75%);

Development • Supplementary information

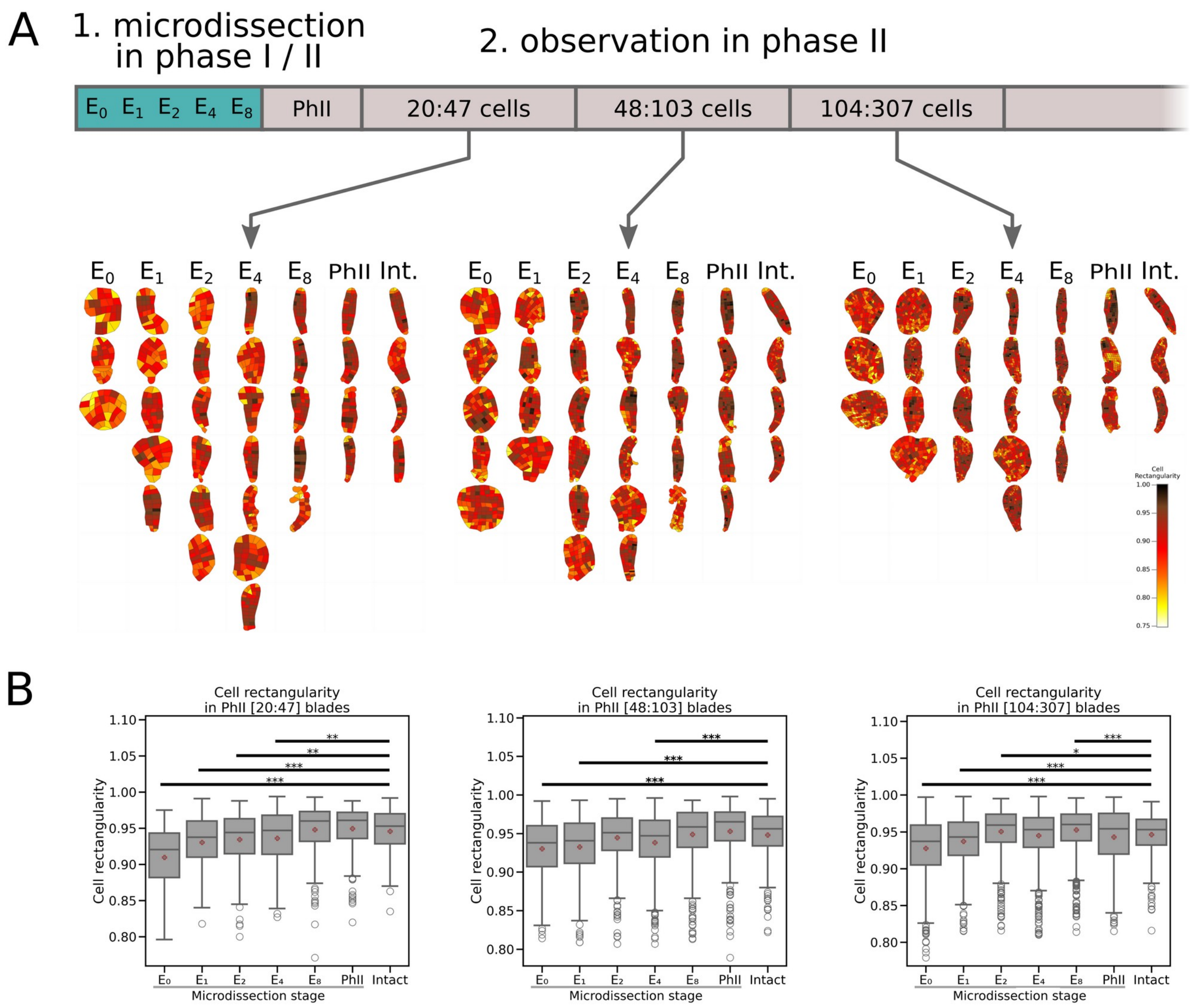

Fig. S6. Impact of severing the embryo from the maternal stalk on embryo cell shape.

(A) Experimental design (see Fig. S3 for details) and heatmap for the cell rectangularity coloured onto segmented embryos at different developmental stages (20–47-cell, 48-103 and 104–307-cell embryos, respectively left, centred and right panels) grown after microdissection of the stalk at E_0, E_1, E_2, E_4, E_8 and PhII stages. Colour scale is indicated on the bottom right-hand side of the figure. The scale is adjusted, with the images of each embryo (blade) all occupying roughly the same space regardless of cell number. (B) Box-plot representation of cell area. The thick red-outlined plus sign in the middle of the box shows the mean; the middle line is the median (2nd quartile); the box includes values between the 1st (25%) and 3rd quartiles (75%); the whiskers show the extent of observations around these values, up to 1.5 times the difference between the 1st and 3rd quartiles; other observations are shown as outliers (open circles). Results of the *t*-test are indicated by p-values: *<5.10^{-2}; ** <1.10^{-2}; *** < 10^{-3}.

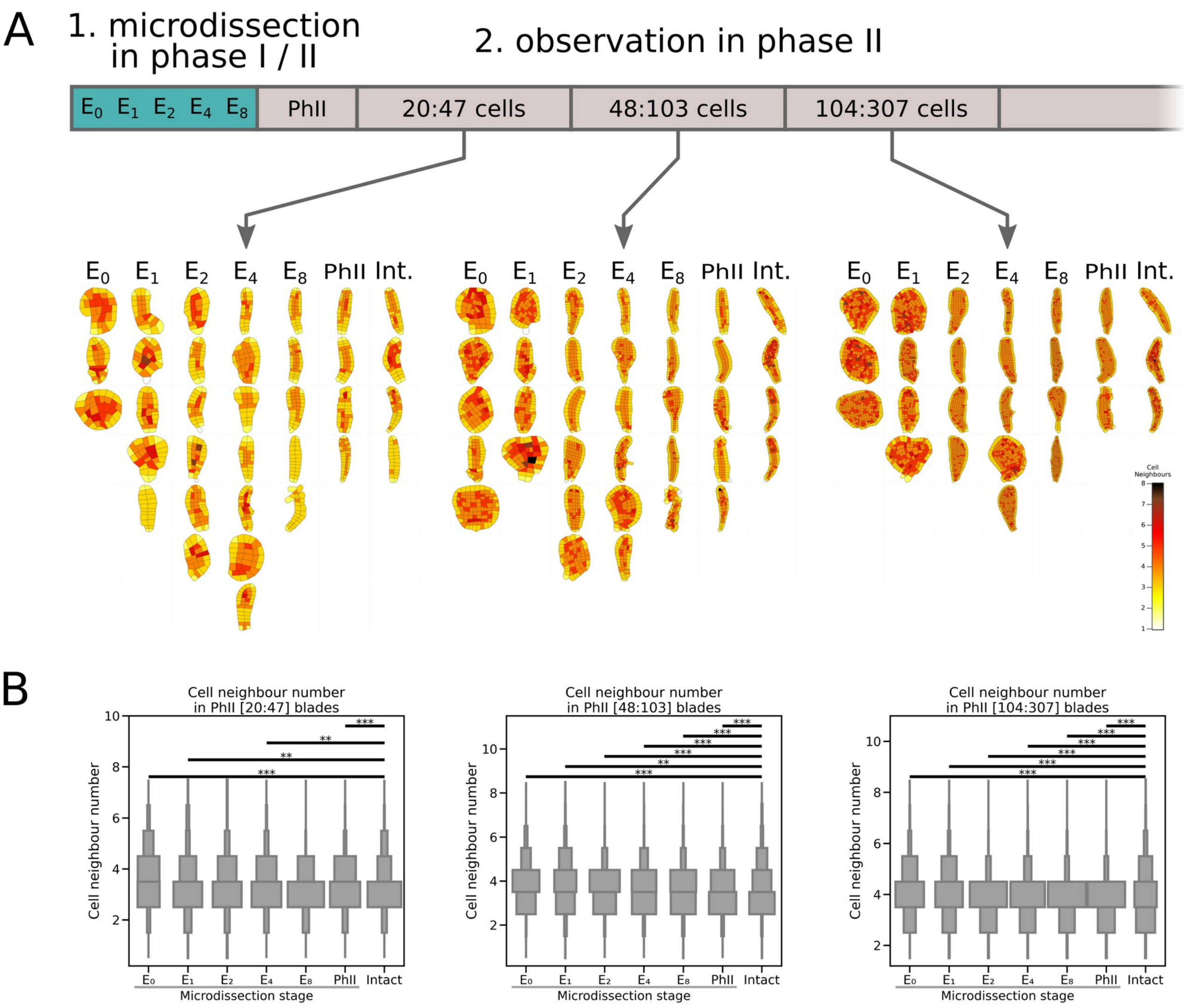

Fig. S7. Impact of dissection of the stalk on the blade topology.

(A) Experimental design (see Fig. S3 for details) and heatmap for the number of cells surrounding one each cell of the embryo (i.e. cell neighbour number), coloured onto segmented embryos at different developmental stages (20–47-cell, 48-103 and 104–307-cell embryos, respectively left, centred and right panels) grown after microdissection of the stalk at E_0, E_1, $E_{2,}$ E_4, E_8 and PhII stages. Colour scale is indicated on the bottom right-hand side of the figure. The scale is adjusted, with the images of each embryo (blade) all occupying roughly the same space regardless of cell numer. (B) Box-plot representation of cell area. The thick red plus sign in the middle of the box shows the mean; the middle line is the median (2nd quartile); the box includes values between the first (25%) and 3rd quartiles (75%); the whiskers show the extend of observations around these values, up to 1.5 times the difference between the 1st and 3rd quartiles; other observations are shown as outliers (circles). Results of the *t*-test are indicated by p-values: *<5.10^{-2}; ** <1.10^{-2}; *** < 10^{-3}.

Development • Supplementary information

Table S1. (S1A) (Left) Counts and (Right) percent of embryos i) growing normally, ii) with a lower growth rate, and iii) with morphological alteration, for stages at which microdissection was performed. The percentage of morphologically abnormal embryos observed in the control (intact) population, due to the morphological plasticity of the genetic strains, is highlighted in blue. (S1B) Probability, calculated from the binomial law, of frequency of embryos with abnormal morphology in the segmented samples (based from observation in populations of intact embryos, Table S1A). From this probability and the size of each sample, the expected number of abnormal embryos is calculated. See Experimental procedures for the calculation of these probabilities.

Available for download at
https://journals.biologists.com/dev/article-lookup/doi/10.1242/dev.202732#supplementary-data

Table S2. Morphometric parameters of the embryos (blades). The table is sub-divided into 3 sections based on the size of the observed embryos (expressed in the number of cells nCells, 1st and 4th columns). “In" are intact embryos. Not all embryos have data available for all three size ranges.

Available for download at
https://journals.biologists.com/dev/article-lookup/doi/10.1242/dev.202732#supplementary-data

Table S3. Cell morphometrics in each embryo (blade). Time point and number of cells are indicated for each embryo. Each cell (one row) was studied for different morphometric parameters. Those relevant for this study are displayed. The data are shown for the three ranges of embryo size (in cell number; e.g. [20:47] cells, indicated by the first column).

Available for download at
https://journals.biologists.com/dev/article-lookup/doi/10.1242/dev.202732#supplementary-data

Development • Supplementary information

Table S4. P-values of statistical tests comparing morphometric parameters characterising *Saccharina* embryos and their cells. *t*-tests were used to compare the cell features, Mann-Whitney tests to compare embryo blades and χ^2 for the analyses of the number of cell neighbours. The quantitative value of each parameter and for each developmental stage at which the stalk was severed (E_n and PhII), is presented as the function of the time window (expressed as the number of cells). In green, p-values lower than 5.10^{-2}. In the main figures presented in the article, only p-values resulting from the comparison between the microdissected embryos and the intact controls are shown.

Available for download at
https://journals.biologists.com/dev/article-lookup/doi/10.1242/dev.202732#supplementary-data

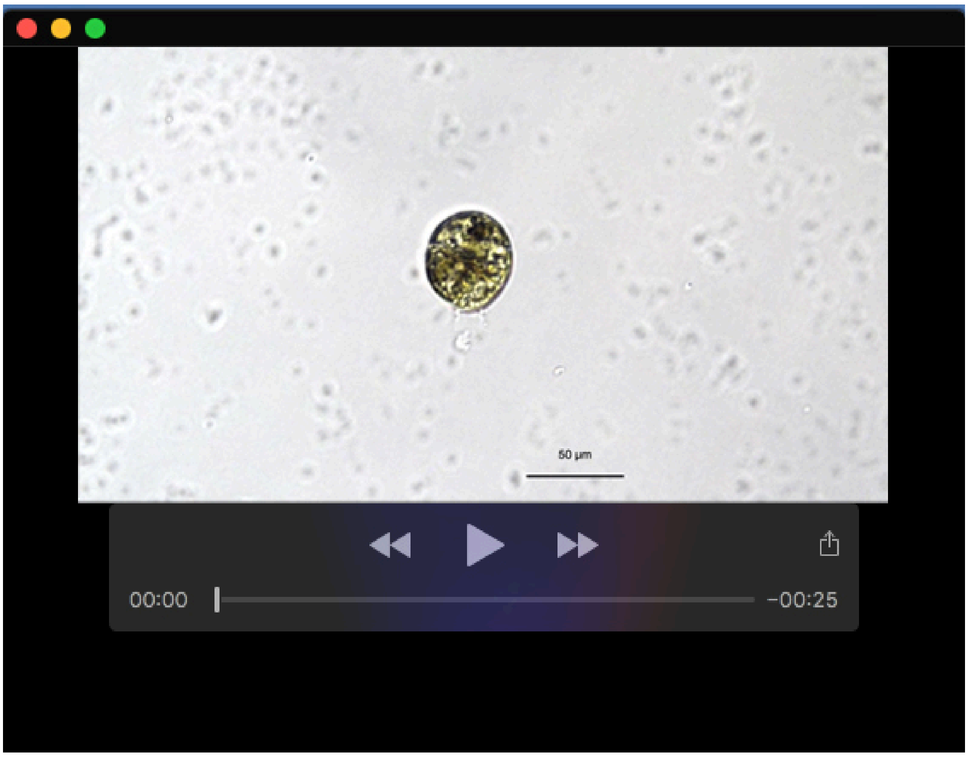

Movie 1. Development (time-lapse) of an early embryo from which the stalk has been microdissected. Leftover pieces (remnants) of the stalk cell wall are visible. Microdissection took place at E_0 (one cell division already occurred before the first time point of the movie).

Development • Supplementary information

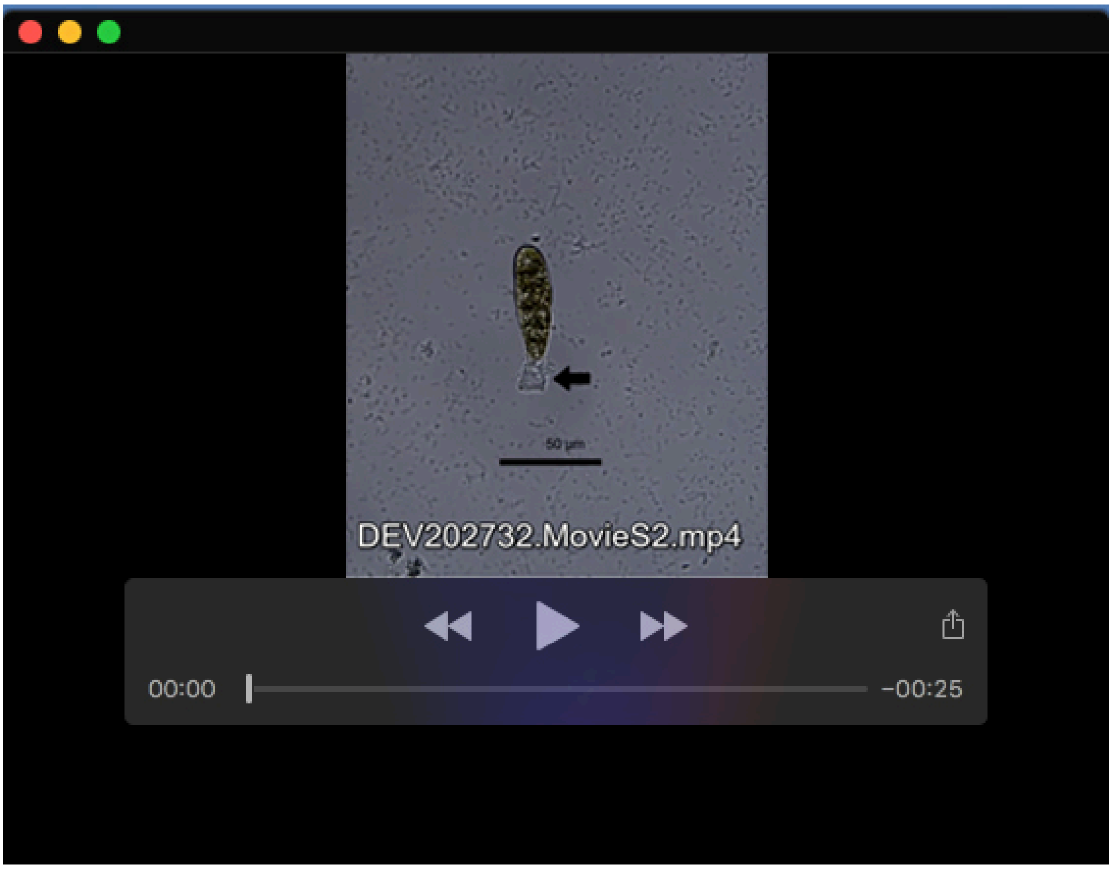

Movie 2. Development (time-lapse) of a 8-cell embryo from which the stalk has been microdissected. Left-over pieces (remnants) of the stalk cell wall are visible.

Development • Supplementary information

Supplementary Materials and Methods

Estimating the risk of spontaneous morphological alteration in a population (intact or microdissected) of F1M1 (female x male) *Saccharina latissima*.

1. Counting embryos displaying a spontaneously altered morphology

In d=3 different Petri dishes containing F1M1 gametophytes, we counted embryos at stage S ≈ 40 cells which either developed with a normal morphology (**N**), or spontaneously displayed an altered (**A**) morphology.

Dish<br>i	Normal (**N**)<br>n_i	Altered (**A**)<br>a_i
2	320	12
3	247	12
4	369	23
Total	936	47

Therefore, noting the dish number by $i=1,\ldots,d$, n_i the number of **N** embryos, a_i the number of **A** embryos, and w_i the total number of embryos in dish i, ($w_i = n_i + a_i$), the weighted mean number of **A** embryos by dish is $\bar{a}=\frac{\sum_{i=1}^{d} w_i a_i}{\sum_{i=1}^{d} w_i}=16.39$ and the weighted variance for a is

$$v=\frac{\sum_{i=1}^{d} w_i \left(a_i-\bar{a}\right)^2}{\frac{d-1}{d}\sum_{i=1}^{d} w_i}=43.52.$$

Development • Supplementary information

The average rate of **A** embryos is $\bar{r}=\frac{\sum_{i=1}^{d} a_i}{\sum_{i=1}^{d} w_i}=0.04781$ with a standard deviation $s=\frac{\sqrt{v}}{\sum_{i=1}^{d} w_i}=0.00671$.

2. Number of spontaneous alterations in samples

When picking one embryo of stage *S* at random, the probability that this embryo develops with an altered shape is $p=\bar{r}\pm s$. For a sample of *n* embryos, the binomial law gives the probability that the number *a* of **A** embryos among these *n* is exactly *k*: $P(a=k)=\binom{n}{k}p^k(1-p)^{(n-k)}$, from which we deduce $P(a\geq k)=\sum_{i=k}^{n} P(a=i)$, the probability to have at least *k* **A** among *n* embryos. The expected number of **A** embryos among *n* is *np*.

With *n*=5, we obtain:

k	low: $p = r - s$		average: $p = r$		high: $p = r + s$	
	P(*a*=*k*)	P(*a*≥*k*)	P(*a*=*k*)	P(*a*≥*k*)	P(*a*=*k*)	P(*a*≥*k*)
0	0.8107	1.0000	0.7827	1.0000	0.7555	1.0000
1	0.1737	0.1893	0.1965	0.2173	0.2178	0.2445
2	0.0149	0.0155	0.0197	0.0208	0.0251	0.0266
3	0.0006	0.0007	0.0010	0.0010	0.0014	0.0015
4	0.0000	0.0000	0.0000	0.0000	0.0001	0.0000
5	0.0000	0.0000	0.0000	0.0000	0.0000	0.0000
Expect	0.2055 **A** in 5 embryos		0.2391 **A** in 5 embryos		0.2726 **A** in 5 embryos	

Development • Supplementary information
