# Supplementary material for: MUM, a maternal unknown message, inhibits early establishment of the medio-lateral axis in the embryo of the kelp Saccharina latissima
Source: Development. 2024 Sep 13;151(20):dev202732. doi: 10.1242/dev.202732 (PMC11423915; doi:10.1242/dev.202732)
Supplement: Supplementary file 1 [file develop-151-202732_review_history.pdf]

MUM, a maternal unknown message, inhibits early establishment of the mediolateral axis in the embryo of the kelp *Saccharina latissima*

Samuel Boscq, Bernard Billoud, Ioannis Theodorou, Tanweer Joemmanbaks, Tanguy Dufourt and Benedicte Charrier
DOI: 10.1242/dev.202732

Editor: Liam Dolan

Review timeline

Original submission:	25 January 2024
Editorial decision:	27 February 2024
First revision received:	4 June 2024
Editorial decision:	17 July 2024
Second revision received:	19 July 2024
Accepted:	30 July 2024

Original submission

First decision letter

MS ID#: DEVELOP/2024/202732

MS TITLE: MUM, a maternal unknown message, inhibits early establishment of the mediolateral axis in the embryo of the kelp Saccharina latissima

AUTHORS: Samuel Boscq, Bernard Billoud, Ioannis Theodorou, Tanweer Joemmanbaks, and Benedicte Charrier

I have now received all the referees' reports on the above manuscript, and have reached a decision. The referees' comments are appended below, or you can access them online: please go to BenchPress and click on the 'Manuscripts with Decisions' queue in the Author Area.

As you will see, the referees express considerable interest in your work, but have some significant criticisms and recommend a substantial revision of your manuscript before we can consider publication. If you are able to revise the manuscript along the lines suggested, which may involve further experiments, I will be happy receive a revised version of the manuscript. Your revised paper will be re-reviewed by one or more of the original referees, and acceptance of your manuscript will depend on your addressing satisfactorily the reviewers' major concerns. Please also note that Development will normally permit only one round of major revision. If it would be helpful, you are welcome to contact us to discuss your revision in greater detail. Please send us a point-by-point response indicating your plans for addressing the referees' comments, and we will look over this and provide further guidance.

Please attend to all of the reviewers' comments and ensure that you clearly highlight all changes made in the revised manuscript. Please avoid using 'Tracked changes' in Word files as these are lost in PDF conversion. I should be grateful if you would also provide a point-by-point response detailing how you have dealt with the points raised by the reviewers in the 'Response to Reviewers' box. If you do not agree with any of their criticisms or suggestions please explain clearly why this is so.

Reviewer 1

© 2024. Published by The Company of Biologists under the terms of the Creative Commons Attribution License (https://creativecommons.org/licenses/by/4.0/).

Advance summary and potential significance to field

This study addresses questions on the early development of polarity in the kelp Saccharina latissima. The subject is a welcome addition to the literature since it addresses important developmental questions in brown algae, a major eukaryotic lineage that is distinct from plants and animals. Such studies are needed to help unify our understanding of multicellular development in poorly understood branches of the tree of life.

During early development in Saccharina, the embryo remains attached to the haploid female gametophyte via an empty cell called the stalk. The authors proceed to dissect early embryos at different stages of development and carefully monitor threir reliance on the stalk for the establishment of polarity in the growing embryo. Apical-basal polarity is important in the Saccharina early embryo since it first develops through a series of longitudinal divisions, before proceeding to initiate transverse divisions later in development.
Through a series of careful observations and tracing the outline of the growing embryos, the authors monitor key metrics like the rate and nature of cell divisions, cell shape changes and cell expansion. Through their experiments, the authors propose that the presence of the stalk at the very earliest stages of development (up to at least the 4-cell stage) is essential to maintain apical-basal polarity, since early dissection results in premature transverse divisions. The authors propose an acronym (MUM – Maternal Unknown Message) as a putative signal that suppresses early transverse divisions in the early embryo. The discussion provides a broad perspective of the implications of their proposed findings.

Comments for the author

Major issue

It is known that many brown seaweeds, including Saccharina, develop a haploid embryo via parthenogenesis from non-fertilised gametes (particularly the female egg cell). It is thus surprising to me why this point is glossed over in the manuscript. In fact, a quick search of the term “parthenogenesis” is not present in their manuscript whatsoever except in one cited reference (Motomura). This issue is crucial to address because the phenomenon they are describing might be conflated by the fact that they are studying parthenogenetic embryos and not true diploid embryos in the detached group. Parthenogenetic embryos and unfertilised eggs are known to detach more easily (see eg. Klochkova et al. 2019) and are known to develop abnormally in kelps like Saccharina latissima (for example reviewed in Bartsch et al; 2008 and Le Gall et al. (1996), who describe these parthenogenetic germlings as “mis-shapen, often consisting of fragile, rounded monostromatic fronds”. These two elements make parthenogenesis difficult to disentangle from the process of detachment and the impact on morphology.

What is the rate of parthenogenesis in their cultures and is it discernible from the detached fraction? This would be easy for the authors to address by counting the frequency of embryonic phenotypes with and without detachment in their unfertilised female gametophyte strain. In Figure 11, the authors show examples of abnormal embryos growing from eggs that were detached from a female gametophyte via mechanical shaking of the petri dish. Does this dish show an unfertilised female? Parthenogenesis could be suggested by the smaller size of the cells as well as their morphology and behaviour, especially in E0 cells that were excised and transferred to another dish (before fertilisation?). These important controls would provide important transparency and ensure that the phenomenon they describe relates to “true” embryogenesis rather than parthenogenesis. In the case of the latter, their data is still valid, but crucial reinterpretation of their findings would be required in light of parthenogenetic development.

Other points:

1. There are multiple instances of “data not shown” throughout the manuscript. As the accompanying statements are often critical to the reasoning, the authors should include this data to ensure transparency. In the age of online publication, is it very easy to provide this data as supplemental information.

© 2024. Published by The Company of Biologists under the terms of the Creative Commons Attribution License (https://creativecommons.org/licenses/by/4.0/).

2. In their measurements of cell growth phenotypes, the authors state that in the absence of the stalk, cells "expand less and maintain an unchanged cell division rate". Is it known whether kelps undergo endoreduplication? In flowering plants, cell expansion is often correlated with increased cell ploidy. If the authors used a DNA stain in their analysis, they could also measure the nuclear:cytoplasmic ratio to further confirm this (although not necessary if their data acquisition did not use such a dye). However, I think it is important to comment on the possibility/knowledge of whether endoreduplication might play a role in cell expansion in Saccharina.
3. The plot in Figure 7A is difficult to interpret and I am not familiar with the format of such a boxplot. I would suggest a clearer plot like stacked barchart or perhaps a cumulative frequency plot. This might help make the differences clearer to see.
4. The images in Figure 2 and 9 are blurry, out of focus and of questionable quality. Some improvements better contrast and a consistent white balance would help interpretation of the images.
5. Figure 8, Figure S1 and Figure S4: While the cell outline drawings are a nice addition, it would help if the lines were darker to help interpretation. The outlines, particularly in the smaller versions, are not very clear in both print and screen view.
6. The method section mentions calcofluor staining and a confocal microscope but it is unclear for what experimental setup this was used as the segmentation was done using bright field images.
7. The authors are somewhat vague on what detachment/excision actually entails. In some places the authors state that the cell wall of the "stalk" seems to be cut with the upper stalk-embryo junction remaining intact, whereas in other cases the treatment is described as a "detachment" from the stalk. From Figure 2, it is not clear whether a (partial) tube is still present.

References:
Le Gall Y, E. A., Asensi, A., Marie, D., & Kloareg, B. (1996). Parthenogenesis and apospory in the Laminariales: A flow cytometry analysis. European Journal of Phycology, 31(4), 369–380. https://doi.org/10.1080/09670269600651601
Bartsch, I., Wiencke, C., Bischof, K., Buchholz, C., Buck, B., Eggert, A., Feuerpfeil, P., Hanelt, D., Jacobsen, S., Karez, R., Karsten, U., Molis, M., Roleda, M., Schubert, H., Schumann, R., Valentin, K., Weinberger, F., & Wiese, J. (2008). The genus Laminaria sensu lato: recent insights and developments. European Journal of Phycology, 43(1), 1–86. https://doi.org/10.1080/09670260701711376

Reviewer 2

Advance summary and potential significance to field

Boscq et al. report on the role played by the stalk cell that anchors the embryo of the brown algae Saccharina latissima to the mother gametophyte in patterning the embryo. The early development of this kelp is characterised by the formation of a stack of up to eight cells before cells rotate their plane of division and divide transverse to form a 2D ovoid blade.
By analysing the consequence of ablating this cell, they conclude that the stalk prevents the premature establishment of the mediolateral axis and promotes cell division and the overall tissue organisation by controlling cell shape. The critical period for the action of the stalk via an elusive diffusible signal dubbed MUM, is until the embryo reaches the 8-cell stage. Without identifying it, the authors set boundary conditions for MUM and its action: it emanates from the stalk cell (a dead cell), does not (or very poorly) diffuse in the medium and requires a physical connection between the embryo and the stalk cell.

Because of their early separation from the plants and animal lineages and the complex shape they acquire, brown algae are exciting new models to study early embryo patterning. This manuscript, like the recent paper of the same group (in Development) that separated the early development of Saccharina into three phases, is very descriptive but essential to set the stage for deeper and hopefully more functional studies soon.

I am very appreciative of the manuscript. The work is meticulous, carefully analysed and interpreted. Most of my comments detailed below have more to do with the way the manuscript is organised and the data presented than the actual experimental results.

© 2024. Published by The Company of Biologists under the terms of the Creative Commons Attribution License (https://creativecommons.org/licenses/by/4.0/).

Comments for the author

1. The manuscript has 11 figures, mostly consisting of two subpanels. I would advise to consolidate these figures into four figures. A possible reorganisation would be: - New fig 2 (effect of detachment at tissue/embryo scale): merge Fig 2 + 3A + 11 (eventually move 3B & 3C to Supp mat).
- New Fig 3 (effect at cell scale): Merge Fig 4, 5, 6, and 7. The heatmaps on 4B, 6B, and 7B feature many samples and could be reduced to show only typical examples, while the complete galleries could be moved to Supp Mat. Fig 8 could be moved to Supp Mat.
- New Fig 4: Merge Fig 9+10.

2. Related to describing the effects at the embryo scale, could the authors perform a statistical morphometric analysis using the set of segmented embryos to display the "average" shape and shape diversity (instead of presenting galleries)?

3. Last paragraph of the introduction: add a few sentences that state the main findings.

4. The two first subheadings of the results, 'Detachment from the stalk impairs both growth and development of the embryos' (l97) and 'Detachment from the maternal stalk leads to a disruption of the body plan and reduced growth' (l121), are redundant. I would only keep one (on l97): "Detachment from the stalk impairs both growth and organisation of the embryos". This section would refer to Fig 1 and the new Fig 2

5. l151: introducing MUM the authors write: "operates in real time". What is meant by this? Clarify or remove.

6. l170: add a comma after MUM

7. l189: tissue topology analysis. The authors write, "Intact embryos were excluded from this quantification because they were segmented manually with a different technique that produced more “broken” outlines and resulted in an overestimation of the number of cell neighbours.". This is critical. How confident are the authors that the difference in the segmentation procedure (that is not described in the methods) is not impacting other aspects (cell size, shape)? The authors should re-analyse the untreated sample with the same methods or provide evidence that the different methods are not introducing any biais.

8. The last section on l275 about abnormal embryos growing from eggs naturally detached from the maternal gametophyte reads ectopic. I wonder if it would not be better to introduce these data at the beginning of the manuscript to draw a parallel between the consequences of the experimental perturbation used (stalk cell destruction) and what can happen in natural conditions.

Reviewer 3

Advance summary and potential significance to field

In the manuscript entitled “MUM, a maternal unknown message, inhibits early establishment of the mediolateral axis in the embryo of the kelp Saccharina latissima”, Samuel Boscq et al. introduced a very interesting phenomenon concerning the effects of the stalk on the establishment of axis in Saccharina latissima zygote. They sectioned the fertilized eggs/zygotes/embryos in different development phases from the maternal tissue with micro-needle, and then observed the development of these detached E/Z/E. They found that the connection to the maternal filamentous gametophyte via the stalk plays key role in the first 4 days after fertilization. After the zygote developed into 8-cell embryo, the detachment would not affect the development anymore. They also performed statistical analysis of morphological changes during the development of the intact and detached E/Z/E.

Comments for the author

The authors provide a wealth of photos of different developmental stages, as well as cell length, width, and area data derived from these pictures. These data have certain reference value for

© 2024. Published by The Company of Biologists under the terms of the Creative Commons Attribution License (https://creativecommons.org/licenses/by/4.0/).

understanding changes in development pattern. They discovered the importance of the connection between the fertilized egg and the maternal filaments for further development, and propose that the attached dead cell contributes to the normal development of the fertilized egg through an unknown message (MUM). The authors tried to identify this MUM, and ruled out several possibilities based on their experimental data. Although the authors discussed multiple possibilities what the MUM could be in the Discussion section, confirming data remains lacking.

In addition, it can be seen from the description in the method section that there are three treated embryos were set as controls, one of which is attached to the bottom of the Petri dish (line 504 ~ 508). However, I couldn’t found in their manuscript that whether these three control groups have different development. Particularly, whether embryos attached to the bottom of the petri dish can develop normally? If so, then what the authors call MUM is unlikely to be a chemical compound(s), but possibly a physical contact that plays a role in determining embryonic development.

First revision

Author response to reviewers' comments

Responses to reviewer

Reviewer 1 Advance summary and potential significance to field

This study addresses questions on the early development of polarity in the kelp Saccharina latissima. The subject is a welcome addition to the literature since it addresses important developmental questions in brown algae, a major eukaryotic lineage that is distinct from plants and animals. Such studies are needed to help unify our understanding of multicellular development in poorly understood branches of the tree of life.

During early development in Saccharina, the embryo remains attached to the haploid female gametophyte via an empty cell called the stalk. The authors proceed to dissect early embryos at different stages of development and carefully monitor threir reliance on the stalk for the establishment of polarity in the growing embryo. Apical-basal polarity is important in the Saccharina early embryo since it first develops through a series of longitudinal divisions, before proceeding to initiate transverse divisions later in development.

-> Comment from the authors: We would like to clarify at that stage of the revision process, that the first series of cell division is transverse, i.e. perpendicular to the longitudinal axis of the embryo, and not longitudinal. This is reported in Theodorou & Charrier, Dev 2023 and in the introduction of our present manuscript.

Through a series of careful observations and tracing the outline of the growing embryos, the authors monitor key metrics like the rate and nature of cell divisions, cell shape changes and cell expansion. Through their experiments, the authors propose that the presence of the stalk at the very earliest stages of development (up to at least the 4-cell stage) is essential to maintain apical-basal polarity, since early dissection results in premature transverse divisions.

-> Comment from the authors: We would like to clarify that the ablation of the stalk results in earlier longitudinal cell divisions (relative to the main axis of the embryo), not earlier transverse divisions. This is also reported in Theodorou & Charrier, Dev 2023 and in the introduction of our present manuscript.

The authors propose an acronym (MUM – Maternal Unknown Message) as a putative signal that suppresses early transverse divisions in the early embryo. The discussion provides a broad perspective of the implications of their proposed findings.

Reviewer 1 Comments for the author

© 2024. Published by The Company of Biologists under the terms of the Creative Commons Attribution License (https://creativecommons.org/licenses/by/4.0/).

Major issue

It is known that many brown seaweeds, including Saccharina, develop a haploid embryo via parthenogenesis from non-fertilised gametes (particularly the female egg cell). It is thus surprising to me why this point is glossed over in the manuscript. In fact, a quick search of the term “parthenogenesis” is not present in their manuscript whatsoever except in one cited reference (Motomura). This issue is crucial to address because the phenomenon they are describing might be conflated by the fact that they are studying parthenogenetic embryos and not true diploid embryos in the detached group. Parthenogenetic embryos and unfertilised eggs are known to detach more easily (see eg. Klochkova et al. 2019) and are known to develop abnormally in kelps like Saccharina latissima (for example reviewed in Bartsch et al; 2008 and Le Gall et al. (1996), who describe these parthenogenetic germlings as “mis-shapen, often consisting of fragile, rounded, monostromatic fronds”. These two elements make parthenogenesis difficult to disentangle from the process of detachment and the impact on morphology.

What is the rate of parthenogenesis in their cultures and is it discernible from the detached fraction? This would be easy for the authors to address by counting the frequency of embryonic phenotypes with and without detachment in their unfertilised female gametophyte strain.

-> Comments from the authors:
We would like to start by bringing some information about the difference between kelp growing as parthenotes and kelp growing from detached eggs. On the one hand, kelp parthenosporophytes can have normal shapes, be either haploid or diploid, and occur at significant, but usually low frequencies, in the natural environment (Goecke et al., Marine Biotech 2022 (doi.org/10.1007/s10126-022-10137-9) and studies on the kelp Lessonia by Oppliger & Correa (J. Phycol 2007 DOI: 10.1111/j.1529-8817.2007.00408.x), have established that parthenogenesis takes place in the field with an occurrence of 2.2%. Their occurrence is calculated from a female-only population and was shown to be lower in female populations mixed with males. It is also calculated based on the presence of the male sex locus (detected by PCR), and not only based on the level of ploidy, because parthenotes can endoreduplicate. On the other hand, the abnormal development of detached eggs has been reported in cultures of Laminariales (Kanda, 1941). These two populations of algal materials, parthenotes and detached eggs, are different in terms of origin and characteristics. Most interestingly here, parthenotes can have normal shapes, while detached eggs are usually malformed.

A) Answers to the question:

1. We did not understand from the cited paper Klochkova et al. 2019 that parthenogenetic embryos and unfertilised eggs detach more easily. Instead, it seems that, once detached, eggs are more difficult to fertilise and, therefore, most detached eggs either die or develop as parthenotes. The fact that they are malformed is not necessarily due to parthenogenesis. However, even if parthenogenetic embryos and unfertilised eggs were to detach more easily, this would have no effect on our observations, because we performed microdissection only on embryos that were initially attached to the gametophyte: the stalk connecting the egg/zygote/embryo to the female gametophyte was manually sectioned and the separated E/Z/E was then immediately transferred into a distinct Petri dish using a glass capillary tube. Consequently, all embryos growing from eggss that detach naturally, parthenogenetic or not, were isolated from the microdissected embryos, because they remained in the original Petri dish with the control embryos. Therefore, there is no possible confusion of the microdissected E/Z/E studied here with embryos growing from naturally detached eggs (parthenogenetic or fertilised).
2. Parthenogenetic/detached (un)fertilised eggs should logically be present in each sample in the same proportions. Therefore, if the presence of these parthenogenetic embryos/unfertilised eggs results in a shift in a given morphogenetic parameter, this shift would occur in all samples. This holds true unless there is a bias towards or against the parthenogenetic embryos/unfertilised eggs in some of the samples. We have no reason to think that there is such a bias.
3. If there were a bias, this bias would be in favour of embryos developing normally, because during the time-course of the experiment, we naturally chose embryos that develop rapidly, in both the intact and the microdissected samples. In addition, the selected embryos (both intact and microdissected) developed up to a developmental stage that allowed us to monitor cell growth and division, as well as embryo shape and lamina topology, i.e. up to the 300-cell stage. Therefore, we preferentially studied the normally growing embryos, not parthenogenetic embryos/unfertilised

© 2024. Published by The Company of Biologists under the terms of the Creative Commons Attribution License (https://creativecommons.org/licenses/by/4.0/).

eggs, which are expected to develop more slowly and/or stop developing at much earlier stage: most of them (90% for our strain) stop growing before the 3-cell stage.

B) Our opinion and position in the revised paper
A recent work deposited in BioRxiv (Dries et al., https://doi.org/10.1101/2024.04.26.591308),) studied the occurrence and morphology of parthenotes in the kelp Undaria, and also to some extent in Saccharina. The aim of our study was to determine to what extent the female gametophyte controls the body planes and cell behaviour in the Saccharina embryo and not to study the occurrence of parthenotes in our cultures. However, whether the phenotype observed in our sample is due to a cause other than the sectioning of the stalk (including parthenotes, but also all other intrinsic causes of malformation, that we can call “morphological plasticity” or “morphological deviation” or “morphological noise” or “intrinsic morphological diversity”) is indeed an important question. Therefore, thanks to Reviewer 1, we now address this issue in the revised version of our paper. To do so, we calculated the ratio of abnormally developing embryos in the intact embryo population, and assumed that this ratio is constant in all embryo populations. Results are shown in Table S1A. Then, we applied this ratio to the population of embryos that have been segmented for our quantitative study. To do so, we calculated (based on the binomial law, explained in the Supplementary Methods) the probability of having “naturally morphological deviant” embryos in our microdissection samples. For example, the evaluated occurrence of having such embryos in the E0 population containing 5 specimens is 0.24 (see Table S1B).

In doing so, we believe we have taken full account of the possible bias in the interpretation of our results due to the presence of ‘intrinsically morphologically abnormal’ embryos (whether they are parthenotes or not).

In Figure 11, the authors show examples of abnormal embryos growing from eggs that were detached from a female gametophyte via mechanical shaking of the petri dish. Does this dish show an unfertilised female? Parthenogenesis could be suggested by the smaller size of the cells as well as their morphology and behaviour, especially in E0 cells that were excised and transferred to another dish (before fertilisation?).

-> Answers from the authors: Figure 11 showed naturally detached eggs. As explained above, naturally detached eggs cannot be present in our samples. We initially showed this figure to compare these eggs with the embryos whose maternal stalk had been severed. In the revised version, we have deleted this figure to avoid confusion, and refer to the observation reported in Kanda (1941) in the introduction instead.

These important controls would provide important transparency and ensure that the phenomenon they describe relates to “true” embryogenesis rather than parthenogenesis. In the case of the latter, their data is still valid, but crucial reinterpretation of their findings would be required in light of parthenogenetic development.

-> Answers from the authors: The point about the occurrence of parthenotes in our sample has been dealt with above.

Other points:

1.There are multiple instances of “data not shown” throughout the manuscript. As the accompanying statements are often critical to the reasoning, the authors should include this data to ensure transparency. In the age of online publication, is it very easy to provide this data as supplemental information.

-> Answers from the authors: Indeed, “data not shown” was mentioned six times throughout the MS (lines 100; 212, 245, 296, 339, 418 of the former version):

Line 107: This reference to unshown data was about the fact that a significant proportion of embryos die immediately or a few hours after the microdissection. We have rewritten the sentence to read: “First, about 16% (at most) of embryos either immediately ceased developing or perished within five days post microdissection”.

© 2024. Published by The Company of Biologists under the terms of the Creative Commons Attribution License (https://creativecommons.org/licenses/by/4.0/).

Line 212 (former version): “Nevertheless, the cells maintain their l/w ratio, at least up to the 100-cell stage (not shown; Suppl. Table 2 for statistical results).” now reads (line 250 revised version): “In summary, in embryos from which the stalk was removed before the 8-cell stage, while nearly all cells maintain their l/w ratio, cells were less rectangular and smaller than in intact embryos.” All the data are in Tables S3 (cell morphometrics) and S4 (result of statistical tests).

Line 245 (former version): The sentence was: “First, we tested whether MUM is a diffusible factor by immersing detached E/Z/E into Petri dishes containing intact fertile gametophytes. We did not observe any modification in embryo development compared with experiments in which embryos were isolated from the maternal tissue and transferred into a Petri dish with fresh seawater (not shown).”

The assessment that the microdissected embryos exposed to intact fertile gametophytes had the same developmental disturbance as those isolated in distinct Petri dishes filled with fresh seawater only (no other algal material was present) was done qualitatively. We did not closely monitor embryo growth using segmentation as we did for the isolated embryos. This would have been challenging because the fertile gametophytes, present in the Petri dish, could have produced additional embryos, attached or detached. We think that, at this stage, a qualitative observation is sufficient to claim that MUM is not a diffusible factor. The sentence now reads (line 292): “By immersing severed E/Z/E in Petri dishes containing intact fertile gametophytes, we tested whether MUM diffuses in seawater. Microdissected embryos growing in the same seawater as intact gametophytes did not develop differently to those isolated in a distinct Petri dish: both groups grew with developmental defaults.”

Line 296 (former version): The sentence: “However, in rare cases, we observed the release of elongated eggs (not shown), suggesting that egg elongation can occur before fertilisation (also reported in Lüning, 1981).” We have one photo of one such elongated egg still enclosed within the stalk, but we decided that this is not the focus of the paper and does not deserve a supplementary figure. Therefore, the sentence has been deleted.

Line 339 (former version): The sentence “Observations show that longitudinal division is less frequent in this region (not shown).” has been deleted because we cannot support this statement with quantitative data.

Line 418 (former version): In the discussion: “Plasmodesmata, which have been observed in both early (up to the 4-cell stage, not shown)”. This sentence has been deleted. In the revised version, we indicate only that these are preliminary data. We have shown that plasmodesmata exists in older embryos in Theodorou & Charrier, Development 2023.

2.In their measurements of cell growth phenotypes, the authors state that in the absence of the stalk, cells “expand less and maintain an unchanged cell division rate”. Is it known whether kelps undergo endoreduplication? In flowering plants, cell expansion is often correlated with increased cell ploidy. If the authors used a DNA stain in their analysis, they could also measure the nuclear:cytoplasmic ratio to further confirm this (although not necessary if their data acquisition did not use such a dye). However, I think it is important to comment on the possibility/knowledge of whether endoreduplication might play a role in cell expansion in Saccharina.

-> Answers from the authors: We showed that, when the stalk is microdissected, the cells are smaller, not larger. Therefore, Reviewer 1 is suggesting that cell size in intact embryos results from endoreduplication, which is inhibited when the stalk is severed, making cells smaller. Endoreduplication in kelps has been addressed by several authors (reviewed in Goecke et al., Front Mar Sci 2020 (doi: 10.3389/fmars.2020.00110). A recent paper from Goecke et al., (Marine Biotech 2022 (doi.org/10.1007/s10126-022-10137-9) shows that, in Saccharina latissima, endoreduplication takes place mainly in the gametophytic phase of the life cycle. Embryonic sporophytes are 2C as expected and nuclei up to 16C are observed only in the medullary cells of older sporophytes. Therefore, the size of intact sporophytes is not related to endoreduplication and severing the stalk cannot negatively control endoreduplication.
We did not address this question in the revised version of the MS.

© 2024. Published by The Company of Biologists under the terms of the Creative Commons Attribution License (https://creativecommons.org/licenses/by/4.0/).

3.The plot in Figure 7A is difficult to interpret and I am not familiar with the format of such a boxplot. I would suggest a clearer plot like stacked barchart or perhaps a cumulative frequency plot. This might help make the differences clearer to see.

-> Answers from the authors: The plot of Figure 7A (now Fig. 3D) can be read like a violin plot. However, a real violin plot is not suitable, because the observed variable is discrete. The legend of Fig. 7 (now Fig. 3D) has been revised to help read the figure: “the width of each box is proportional to the number of cells having the number of neighbours indicated in the Y-axis” has been added (line 706).

4.The images in Figure 2 and 9 are blurry, out of focus and of questionable quality. Some improvements, better contrast and a consistent white balance would help interpretation of the images.

-> Answers from the authors: The images were blurry partly because the figures have been submitted at low resolution to comply with the submission procedure. For the revised version, we have 1) removed Fig. 9 (entitled: “Lack of morphological impact of damaged cells close to the embryo”), which is no longer relevant for the revised version, 2) improved the contrast and the white balance in Fig. 2 (still Fig. 2 in the revised version). However, some photos remain blurry because the embryos tend to grow upright. We tried to select embryos growing horizontally, but this was not possible in all cases. We hope that this does not prevent the readers from obtaining a qualitative overview of the morphological default resulting from stalk removal. Only a quantitative study can provide reliable details on the morphological defaults.

5.Figure 8, Figure S1 and Figure S4: While the cell outline drawings are a nice addition, it would help if the lines were darker to help interpretation. The outlines, particularly in the smaller versions, are not very clear in both print and screen view.

-> Answers from the authors: We thank the reviewer for this comment. In the revised version, we have increased the width of the lines in Fig. 8 (now Fig. 4B) and Fig. S4 (now Fig. S5). However, for Fig. S1, we couldn’t make the lines thicker because the figure displays segmented embryos at different developmental stages. Some have only 2 cells, while others have more than 100 cells. Because all segmented embryos are represented in the boxes of the same size, thicker lines would result in unreadable drawings for the 100-cell embryos. A close-up of Fig. S1 is necessary anyway, because it represents the whole set of segmented embryos. Zooming in allows the lines to be easily seen.

6.The method section mentions calcofluor staining and a confocal microscope but it is unclear for what experimental setup this was used as the segmentation was done using bright field images.

-> Answers from the authors: Calcofluor was used to monitor longitudinal cell divisions shown in Fig. 8 (now Fig. 4B). The legend of Fig. 8 (now Fig. 4B) now indicates how longitudinal divisions are displayed. Line 719: “Longitudinal cell divisions were displayed using Calcofluor staining (see Methods section for detailed protocol).”

7.The authors are somewhat vague on what detachment/excision actually entails. In some places the authors state that the cell wall of the “stalk” seems to be cut with the upper stalk-embryo junction remaining intact, whereas in other cases the treatment is described as a “detachment” from the stalk. From Figure 2, it is not clear whether a (partial) tube is still present.

-> Answers from the authors: Regarding what we named “microdissected E/Z/E”, the stalk was always microdissected with a pulled and sharpened glass capillary tube. As a result, in most cases, a piece of stalk remains attached to the bottom of the E/Z/E. Figure S1 now clearly shows the remnant stalk in embryos of all microdissection stages.

Reviewer 1 may have in mind the work from Bouget et al., Development 1998 and Berger et al., Science 1996, which showed that, in the embryo of the brown alga Fucus, the cell wall of the rhizoid can modify the fate of the thallus cell when it grows in contact with it. This case is different from our study. First, in Saccharina, the embryo cells do not grow in contact with the remaining pieces of stalk. Secondly, before being cut, the stalk was not a tissue, but an empty,

© 2024. Published by The Company of Biologists under the terms of the Creative Commons Attribution License (https://creativecommons.org/licenses/by/4.0/).

dead cell since the extrusion of the egg (the stalk is the cell wall of the oogonium). Therefore, in our study, we aim to assess the role of the stalk as an intact, uninterrupted tube, and the remnants of the stalk at the bottom of the embryo was not addressed. However, how the embryo perceives the stalk is discussed in the Discussion section of our paper.

References:
Le Gall Y, E. A., Asensi, A., Marie, D., & Kloareg, B. (1996). Parthenogenesis and apospory in the Laminariales: A flow cytometry analysis. European Journal of Phycology, 31(4), 369–380. https://doi.org/10.1080/09670269600651601

Bartsch, I., Wiencke, C., Bischof, K., Buchholz, C., Buck, B., Eggert, A., Feuerpfeil, P., Hanelt, D., Jacobsen, S., Karez, R., Karsten, U., Molis, M., Roleda, M., Schubert, H., Schumann, R., Valentin, K., Weinberger, F., & Wiese, J. (2008). The genus Laminaria sensu lato: recent insights and developments. European Journal of Phycology, 43(1), 1–86. https://doi.org/10.1080/09670260701711376

*****

Reviewer 2 Advance summary and potential significance to field

Boscq et al. report on the role played by the stalk cell that anchors the embryo of the brown algae Saccharina latissima to the mother gametophyte in patterning the embryo. The early development of this kelp is characterised by the formation of a stack of up to eight cells before cells rotate their plane of division and divide transverse to form a 2D ovoid blade. By analysing the consequence of ablating this cell, they conclude that the stalk prevents the premature establishment of the mediolateral axis and promotes cell division and the overall tissue organisation by controlling cell shape. The critical period for the action of the stalk via an elusive diffusible signal, dubbed MUM, is until the embryo reaches the 8-cell stage. Without identifying it, the authors set boundary conditions for MUM and its action: it emanates from the stalk cell (a dead cell), does not (or very poorly) diffuse in the medium and requires a physical connection between the embryo and the stalk cell. Because of their early separation from the plants and animal lineages and the complex shape they acquire, brown algae are exciting new models to study early embryo patterning. This manuscript, like the recent paper of the same group (in Development) that separated the early development of Saccharina into three phases, is very descriptive but essential to set the stage for deeper and hopefully more functional studies soon.

I am very appreciative of the manuscript. The work is meticulous, carefully analysed and interpreted. Most of my comments detailed below have more to do with the way the manuscript is organised and the data presented than the actual experimental results.

Reviewer 2 Comments for the author
1. The manuscript has 11 figures, mostly consisting of two subpanels. I would advise to consolidate these figures into four figures. A possible reorganisation would be:
- New fig 2 (effect of detachment at tissue/embryo scale): merge Fig 2 + 3A + 11 (eventually move 3B & 3C to Supp mat).
- New Fig 3 (effect at cell scale): Merge Fig 4, 5, 6, and 7. The heatmaps on 4B, 6B, and 7B feature many samples and could be reduced to show only typical examples, while the complete galleries could be moved to Supp Mat. Fig 8 could be moved to Supp Mat.
- New Fig 4: Merge Fig 9+10.

-> Answer from the authors: We thank Reviewer 2 for these suggestions. We understand that their aim is to group all the data about the embryo lamina together in a single figure, and all the data about the cells together in another big figure. We have considered these suggestions, and have greatly re-organised our manuscript. Below is the list of the main modifications regarding the figures. A complete description of the figure re-organisation follows below. The text has been adapted accordingly (detailed below).

• New Fig. 2 is organised as suggested by Reviewer 2, except for Fig. 11, which has been deleted – it is no longer relevant in the revised version (we do not need to show pictures of detached eggs). Former Fig. 3A is now Fig. 2I. Figs. 3B and 3C have been moved to Fig. S3 (centre).

© 2024. Published by The Company of Biologists under the terms of the Creative Commons Attribution License (https://creativecommons.org/licenses/by/4.0/).

• New Fig. 3 is organised almost as suggested by Reviewer 2. First, we kept a figure showing segmented embryos to illustrate the most important aspect of our study: the first quantitative study of the embryogenesis of a brown alga with complex morphogenesis up to the 300-cell stage (previous detailed studies described the uniseriate filamentous brown alga Ectocarpus and of Saccharina at earlier stages). Thus, the graphs pertaining to cellular features (new Fig. 3B, C, D) are surrounded by images of segmented embryos (Fig. 3A). We think that the readers can better to assess the results in the box plots (showing the means of the quantitative values).

• The graph showing the cell division rate (former Fig. 5) is presented in a distinct figure (New Fig. 4A). It was not grouped with Fig. 3 with all the other cellular parameters, because it was established from another series of time-lapse experiments (shown in Former Fig. S4, now Fig. S5). We have added the former Fig. 8 to this new Fig. 4, because it illustrates the emergence of longitudinal cell divisions. Hence, new Fig. 4 is dedicated to cell division (rate and position).

• Fig 9 “Lack of morphological impact of damaged cells close to the embryo” has been deleted because in the revised version; we now provide more convincing data that the gametophytic cells surrounding the embryos do not impact the establishment of the embryo body plan.

• Fig. 10 is now Fig. 5B.

For more clarity, the correspondence between the former and the revised figures is given below.
Fig. 1: Unchanged
Fig. 2: 2 + 3A
Fig. 3A: 2I
Fig. 3B: Fig S3B middle
Fig. 3C: Fig S3C middle
Fig. 4A: 3B
Fig. 4B: Fig.S4B
Fig. 5: 4A
Fig. 6A: 3C
Fig. 6B: Fig.S6B
Fig. 7A: 3D
Fig. 7B: Fig. S7B
Fig. 8: 4B
Fig. 9: deleted
Fig. 10: 5B (5A: new photos of single-celled gametophyte bearing embryos)
Fig. 11: deleted
added: Fig. S1: photos of microdissected embryos with remaining pieces of stalk
Fig. S1: Fig. S2
Fig. S2: Fig. S3: (+ former Fig. 3B & 3C)
Fig. S3: Fig. S4 (+ Fig. 4B)
Fig. S4: Fig. S5
Fig. S5: Fig. S6 (+ Fig. 6B)
Fig. S6: Fig. S7 (+ Fig. 7B)
none: New Table S1 with occurrence of morphologically abnormal embryos in intact population
Table 1: Table S2
Table 2: Table S3
Table 3: Table S4
None: new Suppl. Methods explaining how the probability of segmenting an embryo with altered morphology arising due to causes other than microdissection of the stalk was calculated.

8. The last section on l275 about abnormal embryos growing from eggs naturally detached from the maternal gametophyte reads ectopic. I wonder if it would not be better to introduce these data at the beginning of the manuscript to draw a parallel between the consequences of the experimental perturbation used (stalk cell destruction) and what can happen in natural conditions.

-> Answer from the authors: We agree with this suggestion. In response to Reviewer 1’s comments, the revised version no longer shows detached eggs. Indeed, our study focuses on severed eggs/zygote/embryos that were isolated immediately after microdissection. Therefore, they cannot be confused with eggs that naturally detach from the female gametophyte when the Petri dish is shaken slightly. We have stressed this point in the Methods sections of the revised version. Line 570: “Note that in our experiment, the occurrence of embryos naturally detached from the maternal gametophytes was excluded, because we manually transferred the sectioned E/Z/E into a new Petri dish.” Consequently, we have deleted Former Fig. 11.

© 2024. Published by The Company of Biologists under the terms of the Creative Commons Attribution License (https://creativecommons.org/licenses/by/4.0/).

2. Related to describing the effects at the embryo scale, could the authors perform a statistical morphometric analysis using the set of segmented embryos to display the "average" shape and shape diversity (instead of presenting galleries)?

-> Answer from the authors: It would be nice to have such a synthetic figure, but it is not possible to display an “average” shape with all the average parameters simultaneously. For instance, the representative embryo of average length and average width, will not have the correct average length/witdh (l/w) ratio. As a hypothetical example, imagine three embryos of length 2, 3, 4 and respective width 1, 2, 3. The average length is l=3, average width is w=2, so l/w = 1.5. However, the l/w ratios of each embryo taken individually are 2, 1.5, 1.33, respectively, giving an arithmetic mean of 1.61 and geometric mean of 1.59. The same holds true for surface area.
Therefore, we do not see a better way to represent shape diversity than showing the observed specimens.

3. Last paragraph of the introduction: add a few sentences that state the main findings.

-> Answer from the authors: We thank Reviewer 2 for this suggestion. The following sentences have been added to the introduction of the revised version: (line 88-91) : “In this way, we show that the physical link between the embryo and the maternal body is essential for the formation of a marked apico-basal axis, which is one of the main characteristics of adult kelps. A negative control of longitudinal divisions appears to be the mechanism through which it is established.”

4. The two first subheadings of the results, 'Detachment from the stalk impairs both growth and development of the embryos' (l97) and 'Detachment from the maternal stalk leads to a disruption of the body plan and reduced growth' (l121), are redundant. I would only keep one (on l97): "Detachment from the stalk impairs both growth and organisation of the embryos". This section would refer to Fig 1 and the new Fig 2

-> Answer from the authors: We appreciate this suggestion, which we have followed in the revised version of our MS. The two main titles of the Results section are now: 1. Severing the embryo from the maternal stalk impairs both growth and the organisation of the embryos, 2. Severing the embryo from the maternal stalk results in altered cell shape and growth. 3. The disruption of the body planes is due to early longitudinal cell divisions in Phase I. 4. MUM is a short-range signal that crosses a dead cell

5. l151: introducing MUM the authors write: "operates in real time". What is meant by this? Clarify or remove.

-> Answer from the authors: The sentence has been replaced by (line 183 of the revised version): “This maternal unknown message (hereafter named “MUM”) is most effective from the egg stage to the 8-cell stage, albeit decreasing in importance with time, and its impact on embryo morphology lasts up to the 100-cell stage.”

6. l170: add a comma after MUM

-> Answer from the authors: Done.

7. l189: tissue topology analysis. The authors write, "Intact embryos were excluded from this quantification because they were segmented manually with a different technique that produced more “broken” outlines and resulted in an overestimation of the number of cell neighbours.". This is critical. How confident are the authors that the difference in the segmentation procedure (that is not described in the methods) is not impacting other aspects (cell size, shape)? The authors should re-analyse the untreated sample with the same methods or provide evidence that the different methods are not introducing any biais.

-> Answer from the authors: We re-segmented the 4 control embryos and the microdissected embryos by the same person. Therefore, for a given shape, the number of segmented lines drawn during the manual segmentation procedure are the same for the microdissected embryos and the controls. Following this re-segmentation of the controls, we re-extracted the morphometric

© 2024. Published by The Company of Biologists under the terms of the Creative Commons Attribution License (https://creativecommons.org/licenses/by/4.0/).

parameters of all segmented images. The corresponding figures have been updated (Fig. 2 for the blade parameters, and Fig. 3 for the cells). The text has been updated, and the sentence “Intact embryos were excluded from this quantification, because they were segmented manually with a different technique that produced more “broken” outlines and resulted in an overestimation of the number of cell neighbours” has been deleted.
In the Materials and Methods section, we have added that the same person performed all the manual segmentation: (line 600): “Z-stack images of time-lapse acquisition were segmented manually by the same person, using Fiji (ImageJ2 version 2.9.0) (Schindelin et al., 2012) …”

*****

Reviewer 3 Advance summary and potential significance to field
In the manuscript entitled “MUM, a maternal unknown message, inhibits early establishment of the mediolateral axis in the embryo of the kelp Saccharina latissima”, Samuel Boscq et al. introduced a very interesting phenomenon concerning the effects of the stalk on the establishment of axis in Saccharina latissima zygote. They sectioned the fertilized eggs/zygotes/embryos in different development phases from the maternal tissue with micro-needle, and then observed the development of these detached E/Z/E. They found that the connection to the maternal filamentous gametophyte via the stalk plays key role in the first 4 days after fertilization. After the zygote developed into 8-cell embryo, the detachment would not affect the development anymore. They also performed statistical analysis of morphological changes during the development of the intact and detached E/Z/E.

Reviewer 3 Comments for the author
The authors provide a wealth of photos of different developmental stages, as well as cell length, width, and area data derived from these pictures. These data have certain reference value for understanding changes in development pattern. They discovered the importance of the connection between the fertilized egg and the maternal filaments for further development, and propose that the attached dead cell contributes to the normal development of the fertilized egg through an unknown message (MUM). The authors tried to identify this MUM, and ruled out several possibilities based on their experimental data. Although the authors discussed multiple possibilities what the MUM could be in the Discussion section, confirming data remains lacking.

-> Answers from the authors: Indeed, not knowing the nature of MUM is frustrating. However, if MUM is a chemical signal, its characterisation requires a much higher amount of material than what we can extract from the stalk, whose volume is estimated to 0.3 10-3 nL. If MUM is mechanical signal, then it is difficult to identify without a genetic approach targeting the genes involved in the control of the cell wall mechanical properties or turgor. Alternatively, shape deformation at the interface of the stalk and the base of the embryos in combination with investigations on Saccharina’s cell wall mechanical properties in this particular region could be informative. Both approaches need years of work and before we can reach that stage, we think that demonstrating that the stalk controls the orientation of cell division is a first necessary step.

In addition, it can be seen from the description in the method section that there are three treated embryos were set as controls, one of which is attached to the bottom of the Petri dish (line 504 ~ 508). However, I couldn’t found in their manuscript that whether these three control groups have different development. Particularly, whether embryos attached to the bottom of the petri dish can develop normally? If so, then what the authors call MUM is unlikely to be a chemical compound(s), but possibly a physical contact that plays a role in determining embryonic development.

-> Answers from the authors: We are not sure that our description of the controls was clear enough for Reviewer 3. First, four controls, corresponding to embryos developing from female gametophytes grown in presence of male gametophytes were used for comparing the embryo and cell morphometric parameters with the microdissected embryos. The female gametophytes are normally stuck to the bottom of the Petri dish and the growing embryos are therefore also held to the bottom of the dish. Please note, however, that they are not directly in contact with the dish, because they are maintained through the stalk only, which is usually upright. These controls were the same for all figures of the MS.

© 2024. Published by The Company of Biologists under the terms of the Creative Commons Attribution License (https://creativecommons.org/licenses/by/4.0/).

The controls to which Reviewer 3 refers correspond in part to former Fig. 9. This part of the study is no longer necessary (see edited text). We have removed this figure and revised the text accordingly. The other controls presented in the Materials and Methods section were not related to this study and we thank Reviewer 3 for pointing out this error.

Second decision letter

MS ID#: DEVELOP/2024/202732

MS TITLE: MUM, a maternal unknown message, inhibits early establishment of the mediolateral axis in the embryo of the kelp Saccharina latissima

AUTHORS: Samuel Boscq, Bernard Billoud, Ioannis Theodorou, Tanweer Joemmanbaks, Tanguy Dufourt, and Benedicte Charrier

I have now received all the referees reports on the above manuscript, and have reached a decision. The referees' comments are appended below, or you can access them online: please go to BenchPress and click on the 'Manuscripts with Decisions' queue in the Author Area.

The overall evaluation is positive and we would like to publish a revised manuscript in Development, provided that the referees' comments can be satisfactorily addressed. Please attend to all of the reviewers' comments in your revised manuscript and detail them in your point-by-point response. If you do not agree with any of their criticisms or suggestions explain clearly why this is so. If it would be helpful, you are welcome to contact us to discuss your revision in greater detail. Please send us a point-by-point response indicating your plans for addressing the referees' comments, and we will look over this and provide further guidance.

Reviewer 1

Advance summary and potential significance to field

As I mentioned in the first round of review, I am in support of this study as I think it addresses an important developmental question in an overlooked branch of eukaryotic life, providing some detailed and compelling data to the community. The authors have addressed the majority of the issues I raised in the first revision, but there are still some sticking points in regards to two points that I discuss in more detail below.

Comments for the author

Parthenogenesis issue In my review of the first version of the manuscript, my main issue revolved around the potential phenomenon of parthenogenesis – the development of embryos from unfertilized eggs – conflating the analysis, which had been glossed over by the authors. Because parthenotes detach more readily and develop abnormally by becoming rounder, parthenogenesis is difficult to disentangle from the process of detachment and its impact on morphology. Parthenogenetic embryos would have likely been prevalent, especially in the early E0 stage and putatively also in E1, E2 fractions, conflating the analysis of true diploid embryos in the detached group.
To address the issue of parthenogenesis in the revised version, the authors have provided additional statistics and in a somewhat convoluted rebuttal, they acknowledge the phenomenon of parthenogenesis in their strain: “(90% for our strain) stop growing before the 3-cell stage”. This statement is missing however in the revised manuscript itself and it would be prudent to add it. The authors calculated the ratio of abnormally developing embryos in the intact embryo population and assumed that this ratio is constant across all embryo populations. They calculated the probability of having “naturally morphologically deviant” embryos in the microdissection samples. For example, the evaluated occurrence of such embryos in the E0 population containing 5 specimens is 0.24 (see Table S1B).

© 2024. Published by The Company of Biologists under the terms of the Creative Commons Attribution License (https://creativecommons.org/licenses/by/4.0/).

It is surprising why the authors use E0 as an example because isn't the E0 "egg" fraction, by definition unfertilized eggs that go on to develop parthenogenetically? Are they fertilised before or after detachment?

The authors even mention in the responses to reviewers: "Instead, it seems that, once detached, eggs are more difficult to fertilize and, therefore, most detached eggs either die or develop as parthenotes." It is very likely that parthenotes develop abnormally at a higher rate than fertilized embryos, as described for other kelp species (Nakahara & Nakamura, 1973; doi.org/10.1007/BF00347797; tom Dieck, 1992; doi.org/10.2216/i0031-8884-31-2-147.1), which this may be linked to the role of sperm centrioles in embryo development (Motomura et al., 1990; doi.org/10.1111/j.0022-3646.1990.00080.x), rather than just the contact with the oogonium stalk. E0 thus appears to be their parthenogenesis control, and would allow them to better compare the morphological defects of detached zygotes with defects induced via parthenogenesis. By acknowledging parthenogenesis in the E0, it would add value to the paper and resolve the issues that this manuscript still faces.
The caption to Figure 3 is also now clarified and states: "This figure demonstrates that the abnormal morphologies of the segmented embryos are due to the sectioning of the maternal stalk, and not to intrinsic morphological plasticity (e.g., potentially due to parthenogenesis)."
In their rebuttal, the authors reason that "there is no link between weaker attachment to the oogonium and parthenogenesis" and that they "did not understand from the cited paper Klochkova et al. 2019 that parthenogenetic embryos and unfertilized eggs detach more easily." To clarify, the Klochkova paper states clearly on page 7: "Many parthenosporophytes of S. angustata that develop from unfertilised eggs do not attach to the oogonium firmly, because cell polarity is disturbed due to a lack of male centrioles (Motomura 1990); thus after they develop a thick cell wall and lose flagella, they fall out of the oogonium. Still, they secrete adhesive glycoconjugates, although not in the basal part, which normally faces the oogonium."

These discussion points are still missing in the revised version. Thus, even though some effort has gone into indirectly addressing some of the issues, the potential of parthenogenesis should be raised in the discussion more clearly in the spirit of transparency.

Detachment issue
This brings me to a second issue that I think still need to addressed as it remains still vague in the revised version - what does detachment/excision actually entail? Figure 2 is still unclear but suggestive of the complete absence of the "stalk" or at least the portion is too small to detect. Although the stalk is persistent (abstract line 24), it is not visible in Figure 2 (except E2, top 2; E4, middle; E8, last one; PhII, first one). On many occasions, there seems to be observable rhizoids, which could be confused with stalks. The authors dp now provide a new figure in Supplement S1 of different eggs, zygotes, and embryos which in most cases have visible pieces of the tube still present (but not for E0), albeit often very small or only the top collar. In their responses to reviewers (point 7), the authors mention: "As a result, in most cases, a piece of stalk remains attached to the bottom of the E/Z/E." Do they thus assume that complete detachment is the same as keeping a bit of the stalk attached? This needs to be explained/discussed more clearly, especially whether/why they expect MUM to be completely absent even if there is a bit of stalk attached. In the methods (ln. 575) they mention a "separation of embryos from their maternal stalk", but this is not really their objective according to the response, where they say they want to disrupt the stalk in the expectation of removing MUM.

The authors mention Berger et al. (1994) Science and Bouget et al. (1998) Development in their response. Moreover, the recent bioRxiv manuscript mentioned by the authors in the responses to reviewers seems to allude that the stalk wall is causal. In view of the mentioned literature, it is important to clarify that such a small piece may not be capable of exhibiting a hypothetical constricting force mediating the basal identity of the attached side or containing enough hypothetical cell fate-determining factors. Therefore, if the population is mixed (with and without pieces of the tube), it is important to be clear on this or to take into account the amount of cell wall material visible in the pictures. It is conceivable that (especially in the young stages) the egg or young zygote might have more easily fallen off when manipulated with the microdissection needle.

© 2024. Published by The Company of Biologists under the terms of the Creative Commons Attribution License (https://creativecommons.org/licenses/by/4.0/).

Reviewer 2

Advance summary and potential significance to field

I thank the authors for the revised manuscript version in which they addressed all the points I raised.

Comments for the author

It occurred that in this revised version, the discussion is very extensive; quasi as long as the result section. Although the points raised are all interesting and relevant, I would suggest the authors to cut the Discussion by at least 1/3. Cross-reading the response to the other reviewers' comments, my attention was drawn to the recent preprint by Dries et al., which investigated a very similar process in Unidaria, converged onto a similar conclusion, and provided hints toward the molecular nature of the signal contributing to correct embryo morphogenesis. It would be appropriate to discuss these data.

Reviewer 3

Advance summary and potential significance to field

I have no further questions.

Comments for the author

No further comments.

Second revision

Author response to reviewers' comments

Reviewer 1 Advance summary and potential significance to field
As I mentioned in the first round of review, I am in support of this study as I think it addresses an important developmental question in an overlooked branch of eukaryotic life, providing some detailed and compelling data to the community. The authors have addressed the majority of the issues I raised in the first revision, but there are still some sticking points in regards to two points that I discuss in more detail below.

Reviewer 1 Comments for the author

General answer from the authors: Parthenogenesis is not the subject of our article. The subject of our article is the role of the connection of the embryo to the maternal tissue. When this connection is interrupted, we have observed altered morphologies and we have studied the possibility that these altered morphologies are due to a reason other than disconnection. The other reasons could be genetic noise, metabolic noise, metabolic differences due to the position of the embryo in the Petri dish, development within a ‘cluster’ of embryos on the same gametophyte resulting in shading effects or spatial ‘crowding’, early or late fertilisation, or no fertilisation (parthenogenesis). Our purpose was not to distinguish between these different sources of noise. We measured the occurrence of altered morphologies in intact control embryos, i.e. still connected to the maternal tissue, but exposed to the same events as those described above (including parthenogenesis), and took the result of this calculation into account when interpreting our data. By doing this, we believe we have fully taken into account the possibility that the altered morphologies are due to a reason other than disconnection from maternal tissue, including parthenogenesis, whereas by focusing solely on parthenogenesis, this would not be the case.
Naturally detached embryos are excluded from our analyses, because we manually transferred the embryos that we microdissected into a separate Petri dish, i.e. away from the rest of the culture,

© 2024. Published by The Company of Biologists under the terms of the Creative Commons Attribution License (https://creativecommons.org/licenses/by/4.0/).

thus excluding the possibility of 'contaminating' the microdissected embryos with any other algal material, including naturally detached embryos.
Furthermore, in both cases, the embryos rarely develop beyond the 3-cell stage, whereas our analysis extends to the 300-cell stage.

Detailed answers are written below.

Parthenogenesis issue
In my review of the first version of the manuscript, my main issue revolved around the potential phenomenon of parthenogenesis – the development of embryos from unfertilized eggs – conflating the analysis, which had been glossed over by the authors. Because parthenotes detach more readily and develop abnormally by becoming rounder, parthenogenesis is difficult to disentangle from the process of detachment and its impact on morphology.

Answer from the authors:
With regard to naturally malformed embryos, whether these embryos are parthenotes is not the point, because i) parthenotes are not especially malformed compared with fertilised embryos (Oppliger et al., 2007; DOI: 10.1111/j.1529-8817.2007.00408.x; Druehl et al. 2005 J Phycol: DOI: 10.1111/j.1529-8817.2005.04143.x) and ii) restricting the calculation of naturally malformed embryos to parthenotes would underestimate the possible bias in our interpretation, because there are other causes of morphological abnormalities. In the previous revised version, we had clarified the distinction between the numerous possible events taking place at that stage: 1) naturally detached eggs (excluded here), 2) naturally malformed embryos (considered here, whatever the cause, including parthenotes), and 3) sectioned E/Z/E, which is the subject of our study (and which may contain "type 2" embryos, the probability of which has been assessed).
In addition, it should be noted that all the causes of developmental malformations, with the exception of the stalk sectioning occur in both control, intact embryos and microdissected embryos. These causes are therefore included in our analyses.

Parthenogenetic embryos would have likely been prevalent, especially in the early E0 stage and putatively also in E1, E2 fractions, conflating the analysis of true diploid embryos in the detached group.
Answers from the authors: We disagree with Reviewer 1. Parthenogenetic embryos are not prevalent in fractions E0, E1 and E2 because 90% of parthenogenetic embryos do not exceed the 3-cell stage. However, our analyses focus on stages of 20 to 300 cells.
To address the issue of parthenogenesis in the revised version, the authors have provided additional statistics and in a somewhat convoluted rebuttal, they acknowledge the phenomenon of parthenogenesis in their strain: "(90% for our strain) stop growing before the 3-cell stage". This statement is missing however in the revised manuscript itself and it would be prudent to add it.
Answer from the authors: Parthenogenesis is not the subject of our paper. The results that we present were not obtained in the context of parthenogenesis. We carried out the experiment mentioned by Reviewer 1 in this review, only because Reviewer 1 asked us for information on parthenogenesis during the previous review.
The authors calculated the ratio of abnormally developing embryos in the intact embryo population and assumed that this ratio is constant across all embryo populations. They calculated the probability of having "naturally morphologically deviant" embryos in the microdissection samples. For example, the evaluated occurrence of such embryos in the E0 population containing 5 specimens is 0.24 (see Table S1B).
It is surprising why the authors use E0 as an example because isn't the E0 "egg" fraction, by definition, unfertilized eggs that go on to develop parthenogenetically? Are they fertilised before or after detachment?
Answer from the authors: Because what we have named E0 develops beyond the 3-cell stage, they are very unlikely parthenotes, as 90% of parthenotes do not develop beyong the 3-cell stage (see above). In addition, Table S1B shows that the probability of having "naturally morphologically deviant" embryos is the same for each stage of microdissection (e.g. E1: E2: E4:). In the text, we have not specifically used the value of E0 but that of samples with 5 embryos (applying to E0, E8 and PhII). This was indicated in the revised version R1: line 150: "Based on the 4.78% of embryos with altered morphology observed in the intact population (Table S1A), we calculated that, in a sample of 5 embryos, the probability of having only normally developing embryos was 0.78, and the expected number of embryos with altered morphology was 0.24 (Table S1B)."

© 2024. Published by The Company of Biologists under the terms of the Creative Commons Attribution License (https://creativecommons.org/licenses/by/4.0/).

The authors even mention in the responses to reviewers: “Instead, it seems that, once detached, eggs are more difficult to fertilize and, therefore, most detached eggs either die or develop as parthenotes.” It is very likely that parthenotes develop abnormally at a higher rate than fertilized embryos, as described for other kelp species.
Answer from the authors: Again, these observations correspond to experiments that go beyond the content of this article, in order to provide some figures on parthenogenesis in our strains. In the microdissection experiments studied in this article, we did not take into account eggs that detach naturally from the stalk and we did not take into account embryos that stop growing after the 3-cell stage. (Nakahara & Nakamura, 1973; doi.org/10.1007/BF00347797; tom Dieck, 1992; doi.org/10.2216/i0031-8884-31-2-147.1), which this may be linked to the role of sperm centrioles in embryo development (Motomura et al., 1990; doi.org/10.1111/j.0022-3646.1990.00080.x), rather than just the contact with the oogonium stalk. E0 thus appears to be their parthenogenesis control, and would allow them to better compare the morphological defects of detached zygotes with defects induced via parthenogenesis. By acknowledging parthenogenesis in the E0, it would add value to the paper and resolve the issues that this manuscript still faces.

Answer from the authors: in our opinion, our MS does not face any issues, at least none related to parthenogenesis, which is fully addressed in the population of “naturally morphologically deviant” embryos.

The caption to Figure 3 is also now clarified and states: “This figure demonstrates that the abnormal morphologies of the segmented embryos are due to the sectioning of the maternal stalk, and not to intrinsic morphological plasticity (e.g., potentially due to parthenogenesis).”
In their rebuttal, the authors reason that “there is no link between weaker attachment to the oogonium and parthenogenesis” and that they “did not understand from the cited paper Klochkova et al. 2019 that parthenogenetic embryos and unfertilized eggs detach more easily.” To clarify, the Klochkova paper states clearly on page 7:“Many parthenosporophytes of S. angustata that develop from unfertilised eggs do not attach to the oogonium firmly, because cell polarity is disturbed due to a lack of male centrioles (Motomura 1990); thus, after they develop a thick cell wall and lose flagella, they fall out of the oogonium. Still, they secrete adhesive glycoconjugates, although not in the basal part, which normally faces the oogonium.”
Comments from the authors: This statement is not based on quantitative data. The term ‘many’ does not even indicate whether it refers to a majority of embryos.

These discussion points are still missing in the revised version. Thus, even though some effort has gone into indirectly addressing some of the issues, the potential of parthenogenesis should be raised in the discussion more clearly in the spirit of transparency.
Answers from the authors: We do not agree with Reviewer 1. Parthenogenesis was discussed in the population of ‘naturally morphologically deviant’ embryos. Parthenogenesis is not the subject of our article. Consequently, there is no reason to focus on parthenogenesis and develop further or this topic.

Detachment issue
This brings me to a second issue that I think still need to addressed as it remains still vague in the revised version - what does detachment/excision actually entail? Figure 2 is still unclear but suggestive of the complete absence of the “stalk” or at least the portion is too small to detect. Although the stalk is persistent (abstract line 24), it is not visible in Figure 2 (except E2, top 2; E4, middle; E8, last one; PhII, first one).
Answer from the authors: The way in which the embryos were separated from the maternal tissue is described in details in the Method section (line 560 of version R1): : “Excision of the maternal tissue at different developmental stages.
The separation of embryos from their maternal stalk was carried out by using pulled glass micro-needles. First, glass micro-needles were prepared by […]. After pulling, the tip of the needle was sharpened to ensure precision cutting. [...] and the tip of the stalk was cut using the glass needle in a cutting motion, while holding the embryo down on the bottom of the dish. The microdissected E/Z/E was transferred into a distinct, new Petri dish, filled with seawater, using a non-pulled capillary and a manual microinjector (Eppendorf CellTram Air 5176). This action was repeated n=100 for eggs, n=100 for zygotes, n=80 for 2-cell, n=80 for 4-cell and n=40 for 8-cell embryos, as well as n=40 early Phase II embryos [...]. Note that in our experiment, the occurrence of embryos

© 2024. Published by The Company of Biologists under the terms of the Creative Commons Attribution License (https://creativecommons.org/licenses/by/4.0/).

naturally detached from the maternal gametophytes was excluded, because we manually transferred the sectioned E/Z/E into a new Petri dish."
In addition, the photos of Fig. 2 are not sufficiently enlarged to allow the remaining stalk to be seen systematically. Therefore, we had added in former revised version a supplementary figure zooming in on the remaining pieces of cell wall (Fig. S1), clearly showing the remaining cell wall at each stage of microdissection, from the E0 (right panel) to the E8.
. On many occasions, there seems to be observable rhizoids, which could be confused with stalks. The authors dp now provide a new figure in Supplement S1 of different eggs, zygotes, and embryos which in most cases have visible pieces of the tube still present (but not for E0), albeit often very small or only the top collar.
Answer from the author: The remaining pieces of stalk are clearly visible in the right-hand panel of Fig. S1. They correspond to sectioned stalk, and we cannot control precisely where the section was made (the length of the rod is about 20 µm).

In their responses to reviewers (point 7), the authors mention: "As a result, in most cases, a piece of stalk remains attached to the bottom of the E/Z/E." Do they thus assume that complete detachment is the same as keeping a bit of the stalk attached?
Answer from the authors: Our study focuses on the physical link with maternal tissue. Therefore, the first objective of our study is to dismantle this link. The photos in Figure S1 show that most embryos have remnants of cell walls. Interestingly, the recent paper by Dries et al. states that the remaining cell walls carry a signal controlling the establishment of body axes locally. Since their conclusion is the opposite of ours, it is crucial to show whether or not the cell wall remains after sectioning of the stalk. Unfortunately, direct comparison is not possible, as the article by Dries et al. does not include photos of the microdissected embryos with remaining cell wall.
This needs to be explained/discussed more clearly, especially whether/why they expect MUM to be completely absent even if there is a bit of stalk attached.
In the methods (ln. 575) they mention a "separation of embryos from their maternal stalk", but this is not really their objective according to the response, where they say they want to disrupt the stalk in the expectation of removing MUM.

The authors mention Berger et al. (1994) Science and Bouget et al. (1998) Development in their response. Moreover, the recent bioRxiv manuscript mentioned by the authors in the responses to reviewers seems to allude that the stalk wall is causal. In view of the mentioned literature, it is important to clarify that such a small piece may not be capable of exhibiting a hypothetical constricting force mediating the basal identity of the attached side or containing enough hypothetical cell fate-determining factors.
Answers from the authors. Our results do not support this hypothesis, as the embryos with pieces of cell wall remaining show an altered morphology.
Therefore, if the population is mixed (with and without pieces of the tube), it is important to be clear on this or to take into account the amount of cell wall material visible in the pictures. It is conceivable that (especially in the young stages) the egg or young zygote might have more easily fallen off when manipulated with the microdissection needle.
Answer from the authors. Our results lead us to conclude that a connection with the maternal gametophyte is necessary for the proper development of the embryo. With or without remaining pieces of stalk. Therefore, for the purposes of this study, it is not necessary to calculate the amount of remaining cell wall.
It seems clear that Reviewer 1 is trying to bring our conclusion closer to that of Dries et al. i.e. that the stalk pieces control the apico-basal axis of the kelp embryo. But this is not what our results say.

*****

Reviewer 2 Advance summary and potential significance to field

I thank the authors for the revised manuscript version in which they addressed all the points I raised.

Reviewer 2 Comments for the author

It occurred that in this revised version, the discussion is very extensive; quasi as long as the result section. Although the points raised are all interesting and relevant, I would suggest the authors to cut the Discussion by at least 1/3. Cross-reading the response to the other reviewers' comments, my

© 2024. Published by The Company of Biologists under the terms of the Creative Commons Attribution License (https://creativecommons.org/licenses/by/4.0/).

attention was drawn to the recent preprint by Dries et al., which investigated a very similar process in Unidaria, converged onto a similar conclusion, and provided hints toward the molecular nature of the signal contributing to correct embryo morphogenesis. It would be appropriate to discuss these data.
Answer from the authors: The revised discussion is now 2596 words long, compared with the previous revision which was 3194 words long (19% shorter). We have 1) avoided repetitions, mainly with respect to our results, 2) deleted the paragraph on organism theory, 3) deleted the paragraph on the relationship between embryo and maternal tissue in animals. We have retained the discussion of 1) the variability of the longitudinal axis displayed by kelp embryos, as this may partly explain the different results obtained by Dries et al, NP2024 (line 229: Therefore, control of Y-axis establishment is a common but plastic trait among individual embryos and among Laminariales species); 2) the possible nature of MUM, for which we refer to the paper by Dries et al. and 3) other cases of physical interactions between maternal gametophytes and embryos in green and red algae, as well as in terrestrial plants. We are sorry not to have been able to reduce it further.

Third decision letter

MS ID#: DEVELOP/2024/202732

MS TITLE: MUM, a maternal unknown message, inhibits early establishment of the mediolateral axis in the embryo of the kelp Saccharina latissima

AUTHORS: Samuel Boscq, Bernard Billoud, Ioannis Theodorou, Tanweer Joemmanbaks, Tanguy Dufourt, and Benedicte Charrier
ARTICLE TYPE: Research Article

I am happy to tell you that your manuscript has been accepted for publication in Development, pending our standard publication integrity checks.

© 2024. Published by The Company of Biologists under the terms of the Creative Commons Attribution License (https://creativecommons.org/licenses/by/4.0/).
